# The Regulation of Cytokine Networks in Hippocampal CA1 Differentiates Extinction from Those Required for the Maintenance of Contextual Fear Memory after Recall

**DOI:** 10.1371/journal.pone.0153102

**Published:** 2016-05-25

**Authors:** Birger Scholz, Amie N. Doidge, Philip Barnes, Jeremy Hall, Lawrence S. Wilkinson, Kerrie L. Thomas

**Affiliations:** 1 Department of Pharmaceutical Biosciences, Uppsala University, Uppsala, Sweden; 2 School of Biosciences, Cardiff University, Cardiff, United Kingdom; 3 Neuroscience & Mental Health Research Institute, Cardiff University, Cardiff, United Kingdom; 4 Schools of Psychology and Medicine, Behavioral Genetics Group, Cardiff University, Cardiff, United Kingdom; 5 MRC Centre for Neuropsychiatric Genetics and Genomics and Institute of Psychological Medicine and Clinical Neurosciences, School of Medicine, Cardiff University, Cardiff, United Kingdom; University of Tasmania, AUSTRALIA

## Abstract

We investigated the distinctiveness of gene regulatory networks in CA1 associated with the extinction of contextual fear memory (CFM) after recall using Affymetrix GeneChip Rat Genome 230 2.0 Arrays. These data were compared to previously published retrieval and reconsolidation-attributed, and consolidation datasets. A stringent dual normalization and pareto-scaled orthogonal partial least-square discriminant multivariate analysis together with a jack-knifing-based cross-validation approach was used on all datasets to reduce false positives. Consolidation, retrieval and extinction were correlated with distinct patterns of gene expression 2 hours later. Extinction-related gene expression was most distinct from the profile accompanying consolidation. A highly specific feature was the discrete regulation of neuroimmunological gene expression associated with retrieval and extinction. Immunity–associated genes of the tyrosine kinase receptor TGFβ and PDGF, and TNF families’ characterized extinction. Cytokines and proinflammatory interleukins of the IL-1 and IL-6 families were enriched with the no-extinction retrieval condition. We used comparative genomics to predict transcription factor binding sites in proximal promoter regions of the retrieval-regulated genes. Retrieval that does not lead to extinction was associated with NF-κB-mediated gene expression. We confirmed differential NF-κBp65 expression, and activity in all of a representative sample of our candidate genes in the no-extinction condition. The differential regulation of cytokine networks after the acquisition and retrieval of CFM identifies the important contribution that neuroimmune signalling plays in normal hippocampal function. Further, targeting cytokine signalling upon retrieval offers a therapeutic strategy to promote extinction mechanisms in human disorders characterised by dysregulation of associative memory.

## Introduction

It is commonly held that the retrieval of a fully consolidated and stored memory by a reminder stimulus can place it in a time-limited labile or plastic phase which may provide a window for updating the memory trace by the incorporation of new information through reconsolidation [1]. Alternatively, cue-associated responses may be overshadowed by the formation of a new memory through a process known as extinction [2, 3].

Fear conditioning in rodents provides an evolutionarily conserved paradigm for investigating psychopathologies involving the dysregulation of aversive memories in humans [4–6]. Such animal studies have suggested that reconsolidation and extinction are distinct entities, as has been argued for consolidation and reconsolidation [7–9]. Distinct conditions of recall initiate either reconsolidation or extinction [10–14] and they are considered to be mutually exclusive processes that may coexist when memory is recalled sequentially [15]. Nevertheless, there appears to be a close connectivity between memory maintenance at recall and extinction since fear memory traces can resurface later after extinction [16], and both require retrieval-initiated labilization processes [17–20]. This suggests the existence of co-dependent regulatory networks for both memory processes. However, not much is known about the regulatory networks, nor how much or little they overlap with each other. A number of proteins and pathways thought to be involved in reconsolidation and extinction have been reported [21–28]. However, a comparison between their molecular signatures is confounded because these reports focus on different specific candidates, use different behavioural settings and measurement parameters. Moreover, there is relatively little information available of how the retrieval stimuli and contextual information more systematically feeds into the gene regulatory networks that underlie the shift to extinction. This calls for a more methodical approach for comparing the molecular signature of extinction with other memory processes.

Interventions that target the post-retrieval memory processes provide a powerful therapeutic strategy for human disorders characterised by dysregulation of associative memory such as post-traumatic stress disorder, drug addiction and schizophrenia [29–32]. Indeed, experimental paradigms of CS exposure that differentially and sequentially engage specific post-retrieval mechanisms have proved to be effective in strengthening the extinction of conditioned fear memory in humans (e.g. [33], though sometimes effects are limited to certain domains of memory, for example emotional but not declarative components [34, 35]. To design effective interventions it is imperative to have a full picture of convergent and divergent molecular and cellular events that underpin consolidation, memory maintenance after retrieval and labilisation, and extinction. Not least, because off-target effects could lead to exacerbating rather than ameliorating pathological memory. For example, D-cycloserine has been reported to act as both a reconsolidation-blocker and extinction-enhancer in rodents depending on the conditions of recall (Lee, 2009).

Here using Affymetrix GeneChip Rat Genome 230 2.0 Arrays, we investigated the CA1 transcriptome changes after the selective engagement of extinction of contextual fear memories (CFM) in the rat by a single prolonged recall session. In order to provide a more extensive analysis of the gene regulation underlying extinction with other memory processes, using the same systematic analytical approach we re-examined previously acquired Affymetrix GeneChip transcriptome changes associated with consolidation after contextual fear conditioning (CFC) and those initiated by a single short conditioned context re-exposure previously associated with reconsolidation [36]. We now show that the transcriptome profile of extinction in CA1 is distinct from the profiles initiated by a short context re-exposure at recall and CFC. Whilst recall *per se* regulates genes associated with immune responses, extinction involved fewer genes of this category and those involved had different identities compared with recall that does not produce extinction. An *in silico* phylogenetic footprinting and chromatin immunoprecipitation approach also found the promoter structures associated with the transcriptome profiles to be generally distinct from each other and identified transcription factor NF-κB signalling dissociated between reconsolidation-attributed and extinction networks. Our report provides direct evidence that that the transcriptional regulation of NF-κB differs between the two post-retrieval processes [37, 38] and identifies the novel target molecular networks affected.

## Material and Methods

**Fig 1:**
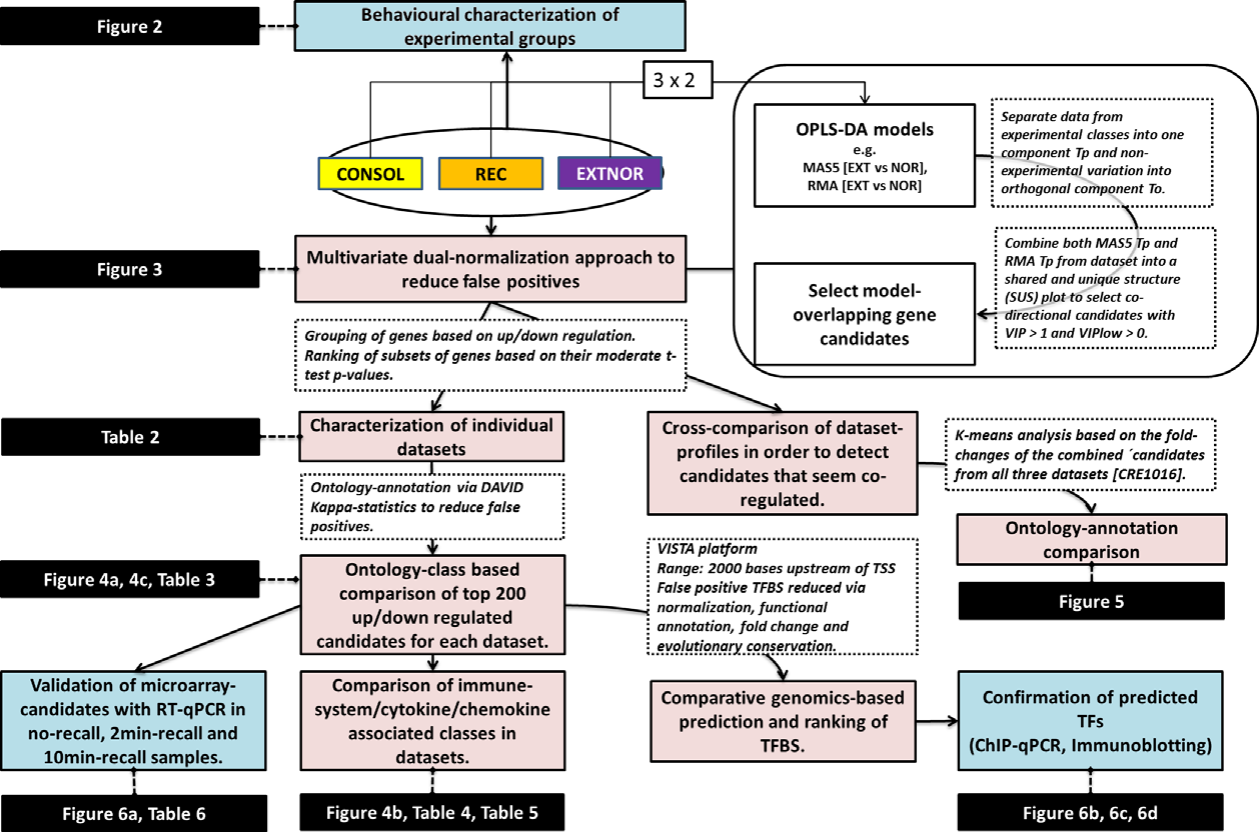
Flow-chart of methods and data analysis. The three different datasets (CONSOL, REC, EXTNOR) are analysed and filtered down to number of different gene candidate subsets based on normalization, fold change directionality, ontological annotation and over-representation analysis, and evolutionary conservation of promoter structures. Links to the different figures and tables are included in the black boxes.

A general flow chart of the methods and data analysis used in this study is shown in Fig 1.

### Animals

Adult male Lister hooded rats (280–350 g) were housed in pairs in holding rooms maintained at a reversed-light cycle (12 h light/dark). Food and water were freely available. All experiments were conducted in the dark period. Male rats were used to minimise the variance on CFC performance and extinction by the oestrus cycle [39]. Nocturnal rats were trained and tested at the same times within in their active phase because of the circadian influence on gene expression and brain plasticity [40]. Thus the regulation of gene expression associated with the experimental manipulations was less confounded by extraneous sources of variation. After completion of the behavioural procedures, all animals were sacrificed by carbon dioxide inhalation. Experiments were conducted in accordance with the United Kingdom 1986 Animals (Scientific Procedures) Act (Project license PPL 30/2722).

### Behavioural Contextual Fear Conditioning (CFC) Procedures

**Fig 2:**
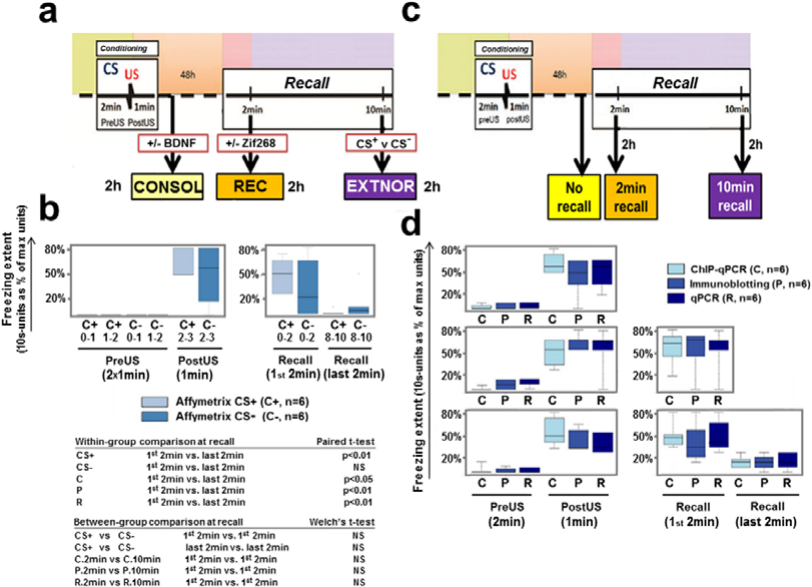
Behavioural data. **a)** Schematic overview of experimental behavioural protocols used to generate the CONSOL, REC, and EXTNOR microarray datasets. **b)** Conditioned freezing behaviour in animals used to generate the EXTNOR Affymetrix dataset. Freezing behaviour was assessed once every 10s. The extent of freezing is shown as number of 10 s interval units converted to percentage of max number interval units (max = 6 units for 1 min, max = 12 units for 2 min). The box plots show the median values and upper and lower limits of the boxes are the upper and lower quartiles. The whiskers show the max and min values. As the max time differs between PreUS (2 min) and PostUS (1 min), the PreUS is plotted by two boxplots (0–1 min and 1–2 min) and the y-axis of the PreUS/PostUS plots represents a max of 1 min (i.e. 6 x 10 s units). The Recall box y-axis represents a max of 2 min (i.e. 12 x 10 s-units). **c)** Overview of experimental design for follow-up studies with qPCR (R), immunoblotting (P) and ChIP-qPCR (C). **d) and b)** Behavioural characterization of the rats for follow-up validation assays. The three rows of results represent the data from the No Recall, 2 min and 10 min recall groups, respectively. Statistical significance was tested for within-group differences (2 min vs. 10 min, paired t-test) and between-group differences between the 2min groups and 10min groups (2 min vs. 2 min and 2 min vs. 10 min, Welch’s t-test).

For each experiment in the study, one operator processed all animals, the behavioral and experimental manipulations were carefully time-locked, and the handling of individual animals in each group was ordered pseudo-randomly. For the microarray analysis, rats were first pre-exposed for 3 d to two experimental chambers (Med Associates Inc., St Albans, VT) for 10 min/d. These contexts were designed to differ in a number of features including size, spatial location, odor, and lighting. In addition, to further distinguish the two contexts, exposure to each chamber was separated by a minimum of 4 hours. The conditioning trial was given 24 hours later (Fig 2). Conditioning consisted of the rats being placed individually in one of the chambers for 3 min in a counterbalanced manner. After 2 min a single scrambled foot shock (US, 0.5 mA for 2 s) was delivered. Rats were either returned to the CS conditioned context (CS+, EXT group) or to the other (CS-, NOR group) context for 10 min, 48 h after fear conditioning. All rats were housed in their home cages out side the training and testing sessions. In the follow-up studies for qPCR, ChIP-qPCR and immunoblotting, animals were conditioned in one context and either allowed to continue in the home cages for 48 h (*No recall* (NR) group), or returned to the conditioning chamber for either 2 min (*2min recall* (2R) group) or 10 min (*10min recall* (10R) extinction group). Animals in the follow up studies did not receive context pre-exposures prior to CFC. Animal behavior was electronically recorded and their extent of freezing quantified by an observer blind to the experimental group. One unit of freezing was defined as an absence of movement other than that required for respiration, once every 10 s. Extent of freezing was expressed at the percentage of time spent freezing in the pre-US and post-US epochs during CFC, and for the first and last 2 min (in the case of prolonged 10 min context exposure) of the recall trial. Paired t-tests were used to analyse within-session and between-session changes within experimental groups and Welch two sample t-tests for between-group comparisons (α = 0.05, unless specified). All data analysis on behavioral data and otherwise was conducted with the R software (version 2.13.2).

### Tissue collection

Immediately after sacrifice, the dorsal hippocampal CA1 region was collected through microdissection on ice. For the microarray work, Western blotting, and ChIP-qPCR, the tissue was snap-frozen on dry ice and stored at -80°C before further sample preparation. CA1 tissue was immersed in Ambion RNAlater (Invitrogen Ltd, Paisley, UK) for gene expression analysis by qPCR.

### Microarray hybridization

Tissue was collected two hours after the behavioural tests. RNA sample preparation and microarray hybridization was conducted as previously described [36]. Total RNA was isolated with RNeasy (Qiagen Ltd, Crawley, West Sussex, UK, 8.14 +/- 0.44 μg total RNA per dorsal CA1) from individual animals. RNA quality was assessed spectrophotometrically on an Agilent Bioanalyser (Agilent Technologies UK Ltd, Stockport, Cheshire, UK), and only samples with a RNA Integrity Number (RIN) of 8/10 or more went forward for hybridization to Affymetrix GeneChip Rat Genome 230 2.0 Arrays (31,042 probe sets/gene representations, Agilent Technologies UK Ltd, Stockport, Cheshire, UK) according to the manufacturer’s protocol at the Central Biotechnology Service, Cardiff University, UK. No amplification steps were performed before cDNA synthesis before hybridization because RNA extracted from the individual CA1 samples met the 1–5 μg total RNA requirements for the reverse transcription step and subsequent hybridization to the Affymetrix array. The oligonucleotide microarray slides were scanned with an Agilent microarray scanner. Each individual RNA sample was hybridized to a separate array.

### Microarray analysis

Alongside the new hippocampal CA1 extinction Affymetrix datasets (EXTNOR, EXT n = 6, NOR n = 6), we also reanalysed Affymetrix datasets previously reported to be associated with consolidation (CONSOL) and reconsolidation (REC, *which we will now refer to as recall*) [36]. The CONSOL datasets represent gene expression in dorsal CA1 two hours after CFC in conditions where either antisense targeting BDNF (BDNF-ASO, n = 8)) or scrambled BDNF missense sequence (BDNF-MSO) was infused into the hippocampus 90 min prior to conditioning. The REC datasets represents expression in the dorsal CA1 two hours after a short 2 min CFM retrieval trial two days after CFC. The two REC datasets result from gene expression measured in rats that had received intrahippocampal infusions of either antisense targeting of Zif268 (Zif268-ASO, n = 8) or a missense sequence (Zif268-MSO, n = 8) 90 min before CFM retrieval (Fig 2A). The MSO datasets for CONSOL and REC correspond to gene expression associated with the consolidation and recall of CFM, whereas the ASO datasets act as discriminator groups since the BDNF-ASO and Zif268-ASO respectively impair these two processes following CFC and recall [7] by attenuating BDNF-mediated protein synthesis necessary for associative plasticity[41] or by reducing the availability of the transcription factor Zif268 directly. It should be noted that Zif268-ASO impaired the subsequent expression of CFM only when administered prior to recall and thus de facto the labilised memory. Over 80% of the candidates from the CONSOL and REC gene sets reflect gene transcripts truly regulated following the acquisition or retrieval of CFM in models not dependent on ASO infusions[36]. Thus, all datasets were generated from dorsal CA1 tissue two hours after either CFC, or two hours after a brief (2 min) or prolonged (10 min) exposure to a context (conditioned or non-conditioned) two days after CFC (Fig 2A). Our general strategy was to constrain our within-experiment design to generate candidate lists that were enriched for genes necessary for consolidation, recall or extinction rather than all genes regulated by the unique behavioural conditions used to initiate each process. Therefore, by reducing the probability of measuring gene expression associated with experiential modulatory factors, such as activity or exposure to stressors which differ across behavioural paradigms, we increased the probability of identifying common pathways regulated in associative memory processing and enhanced the validity of comparisons between the candidate lists. Note that this experimental approach will select for a subset consolidation and reconsolidation-attributed genes and is likely to exclude genes regulated independently of BDNF and Zif268. However, those missing from the analysis are likely to be proportionally small in number because:—(1) nearly 1000 candidate genes were identified with each of the two memory processes, (2) in the absence of BDNF-dependent signalling the conditions for consolidation are not met[7], and (3) blocking the activity of a transcription factor suggested to be crucial for reconsolidation is likely to impair the expression of a large number of genes that are regulated by the complex interplay of transcription factors which involve Zif268. Therefore, our data sets are likely to be enriched and representative for genes regulated in the two memory processes.

### Transcriptome data analysis

The data analysis was designed to identify systematic trends in the different data sets in a manner that reduced the overall bias introduced by singular normalization algorithm choice. To this end, the data was analysed using a combination of univariate and multivariate approaches. The raw intensity data from all three experimental Affymetrix data sets (CONSOL, REC, and EXTNOR) were normalized using MAS5 and RMA (MAS5 alone having previously been used for the analysis of CONSOL and REC [36]) to generate two datasets). Univariate analysis was conducted on each of the six datasets using the lmFit() Limma function [42]. Empirical Bayes moderated t-test statistics were calculated for the pair-wise comparison within each experiment. In order to provide an initial broad scale classification and ranking of between-group and within-group variation differences, pareto-scaled orthogonal partial least square discriminant analysis (OPLS-DA, see also [43]) together with a jack-knifing based cross-validation was performed (Umetrics, SIMCA-P v.13). Within each experimental dataset two OPLS-DA models were created: one for each normalization type. Shared probes between models were selected (CONSOL n = 1150 (C1150), REC n = 964 (R964), EXTNOR n = 969 (E969)) using a combination of OPLS-DA loadings. Probes with |Cor (Tp, X) within a radius of r = 0.75 from the edge origo in a scaled scatter plot and the virtual importance of the projection (VIP) > 1.0 with the lower VIP confidence interval limit ≥ 0.0 were chosen. Within-experiment enrichment overrepresentation analysis was conducted on the top 200 up- and down-regulated ranked probes using the sum of the two moderated t-test p-values (from lowest to highest) in each OPLS-DA derived dataset (C1150: C200mso and C200aso, R964: R200mso and R200aso, E969: E200ext and E200nor). Within experiment enrichment overrepresentation and ontology term cluster analysis was conducted through the DAVID platform [44] using the annotated probes in the Affymetrix Rat Genome Array 230.2 as background, and the modified Fischer exact score EASE p < 0.01 as cut-off.

Enrichment analysis was conducted against Gene ontology biological process (GOBP), cellular compartment (GOCC), molecular function (GOMF), KEGG, Panther biological process (PBP), Panther Molecular function (PMF) and Panther Pathways (PPW). Kappa statistics (Kappa threshold 0.7) were used to measure the degree of the agreement in sharing of genes between annotation terms. Between-experimental condition cluster analysis was conducted using k-means (n probes = 1016, k = 6, 1000 random starts) for all probes that where up- and down regulated on the MAS5 and RMA based log2 fold changes, and VIP and OPLS-DA top-ranked in at least one out of the three candidate sets (C1150, R964 and E969). Fold-change profile clusters with the same general silhouette and gene content between MAS5 and RMA-based fold change values were chosen for enrichment analysis (using the same parameters in the DAVID platform as described above). Heat map visualizations of number of genes per ontology terms were conducted using complete linkage hierarchical clustering.

### Promoter analysis

Probes among the top candidates orthologous to mouse and human were used for phylogenetic footprinting to identify potential transcription factors (TFs) regulating the observed gene expression in the different datasets. Using rVISTA and PROLOGAN for alignment [45], TF binding sites were considered potentially conserved and significantly enriched in orthologous candidate genes if within 2000 bases from the transcription start site (TSS) p < 0.0005 when compared to the rest of the genome. Individual genes were then followed up more in depth in the rat genome using the VISTA platform [46].

### Quantitative PCR analysis

Using the QIAGEN RNeasy kit, RNA was isolated from CA1 tissue from individual animals (n = 6 for each the control *No recall*, *2min recall* and *10min recall* groups). cDNA synthesis was performed using the GoScript™ Reverse Transcription System (Promega). qPCR was conducted with FastStart Universal SYBR Green Master (Roche) on the Stratagene MX-3000P (Agilent Technologies; 1 cycle 95°C, 10 mins; 40 cycles of 95°C, 1 min; 60°C, 1 min; 72°C, 1 min). All qPCR samples were run in duplicate and the outcome was calculated using 2 ΔΔCt method (Table 1 for primer sequences). Statistical significance was calculated by comparing the *2min* and *10min recall* groups against the *No recall* group (Dunnett’s test).

**Table 1:** List of primers used in the RT-qPCR and ChIP-qPCR experiments.

Experiment	Gene	Forward	Reverse
RT-qPCR	*Ahnak*	GATGTGGACATGTCTCTTCCC	AGGCTCACATCCACTTCAGG*O*
	*Camk2n1*	GCCATGTCCGAGATCCTAC	GGTTGCCAGCGAAGAAGG
	*Cldn5*	GCCTTGGTGCTGAGTACTTG	GTTCGCCAACATCGTAGTCC
	*Cldn11*	TGACCTGCAGCTACACCATC	GCAATCATGAGGGCTCTACAA
	*Cxcl1*	AGGGCGGAGAGATGAGAGTC	AGGCATTGTGCCCTACAAAC
	*Ccl2*	CAGTTAATGCCCCACTCACC	TTCCTTATTGGGGTCAGCAC
	*Gapdh*	GAGAAACCTGCCAAGTATGATGAC	TCCACCACCCTGTTGCTGTAG
	*Gli1*	AGGGGCAGGATAGGAGACTG	AGCAGGAATTGTTGTGGGAG
	*Gli2*	AGTACAGACGCTGCATGTGG	TGGGGTTTCTGTGGACTAGG
	*Gli3*	ACTACGCTTTTCCACGGATG	AGATGCAGTGATGGAGGAGG
	*Icam2*	GATAAAGCAAAGCAGGACGG	GCCATATTCAACAGCACAGC
	*Il1a*	CTGAAGAAGAGACGGCTAAG	ATGATGAACTCCTGCTTGAC
	*Nfkbia*	CATCTCCACTCCGTCCTG	GCACCCAAAGTCACCAAG
	*Nfkbiz*	CCGTTGATGGTTCTTTGGTC	TGCTTTGAGGGTGCCTAATC
	*Ocln*	TCCAGTTCTCTGTCGAGACG	CCCAGACCACTATGAAACCG
	*Opalin*	AGAAGAACCACCGATGAGCC	GGCTCCTCACTTCCTGAGAC
	*Rara*	GTCTTCATCACCAGCAAAC	ATCTTCTGTCACCATCCG
	*Rela*	AGCTTCTGAACCAGGGTGTG	AGCTGAGAAGTCCATGTCCG
	*Sucla2*	GGCTTCGGGTATTAATGTGC	ATGGCTGCAAGAAACGAGAC
	*Ywhaz*	CAATCAACGGTTGCCATAGC	GGGTTTCCTCCAATCACTAGC
ChIP-qPCR	GLI1 on *Cldn5*	GCTGTACCTCGCCATCCTAC	CCTAACCCAGTGGACCTTTC
	GLI1 on *Gli2*	GCTGACAGGGCTGACAGACT	CCTAAGCAGATGTCCCCAAA
	NFκBp65 on *Il6*	ATGAGCTACAGACATCCCCAGTC	CCTCCTAGCTGTGATTCTTTGGA
	NFκBp65 on *Nfkbia*	GCGAGTTCAGACTGTTGTGG	GGGTTTAGGCTTCTCAGTCG
	NFκBp65 on *Nfkbiz*	AAACAGCTTTGTGGGGCTTC	CTATGCTCATCCTCGGGTTC
	NFκBp65 on *Rara*	CGGACCTCCTGTTCCTAGTC	AACGTCTCCACCTTCAGCAC

### ChIP-qPCR analysis

Chromatin immunoprecipiation (ChIP) was performed on CA1 samples from individual rats using the bead-based LowCell# ChIP kit (Diagenode). Optimal ChIP conditions were identified with regular ChIP-PCR (data not shown) leading to the use of 18 sonicator pulses (Bioruptor, Diagenode) with 470 μl Buffer B Solution, and 3 μg of antibodies. The antibodies used were GLI1 (cat#2553, Cell Signalling Technology), NF- κBp65 (cat#3034, Cell Signalling Technology) and IgG (cat#kch-819-015, Diagenode). ChIP-qPCR was conducted with FastStart Universal SYBR Green Master (Roche) on the Stratagene MX-3000P (Agilent Technologies; 1 cycle 95°C, 10mins; 45 cycles of 95°C, 1 min; 60°C, 1 min; 72°C, 1 min). Primers were designed using USCS Genome Browser and Primer3 (Table 1). All ChIP-qPCR samples were run in duplicate and the outcome was analysed by calculating the percentage of ChIP-input to genomic input (0.25% concentration) and comparing the *No recall*, *2min* and *10min recall* groups against the negative IgG control using Dunnett’s test.

### Immunoblotting

Protein sample preparation was conducted by bead-homogenization (Fast-Prep-24system, MP Biomedicals) for 4 x 20 s in 8M Urea Buffer (8 M urea, 4% CHAPS, 40 mM Tris base, 1% DTT). Protein concentrations were determined using the 2-D Quant Kit (GE Healthcare). For GLI1 analyses, 30 μg proteins per sample were loaded and separated on 5% SDS-PAGE gels (120 V, 120 mins; XCell blotting tank, Invitrogen), then transferred to nitrocellulose membranes (Invitrogen) for blotting (Trans-Blot Cell, BIO-RAD; 30 mA, overnight) using a 10% MeOH and 0.5% SDS Towbin transfer buffer. Blots were blocked in TBST (5% non-fat milk powder, 0.01 M Tris-buffered saline, 1% Tween 20). For NF-κB-p65, 30 g proteins were separated on 10% SD-PAGE gels (120 V, 120 mins) before blotting (90 V, 100 min) using a 20% Towbin transfer buffer. Primary antibodies (GLI1, cat#2553, Cell Signalling Technology; NFKBp65 cat#3034, Cell Signalling Technology; alpha-Tubulin cat#926–42213, LI-COR) were used at the following concentrations: alpha-Tubulin, 1:2000; NF-κBp65, 1:500; GLI1, 1:500. Secondary antibodies were applied at 1:10,000 (goat-anti-rabbit IRD 680RD, cat#926–68071, LI-COR; donkey anti-mouse IRD 800CW, cat#926–3221, LI-COR). Western blots were scanned using the Odyssey CLx system (LI-COR). Intensity values were normalized against local background and alpha-tubulin levels (Odyssey software, LICOR). Western data was compared against each other using Students t-test for No recall vs. 2 min recall within one gel run/blot, and 2 min vs. 10 min within one gel run/blot.

## Results

### Behaviour

Rats demonstrated a clear increase in freezing behaviour after the US during fear conditioning (PreUS vs. PostUS, Fig 2B and 2D). Re-exposure to the conditioned context (CS+) but not a familiar, non-conditioned context (CS-) for 10 min resulted in the retrieval of the CFM and a reduction in CS+ associated conditioned freezing during a subsequent test (S1 Fig). Thus re-exposure to a CS+ but not CS- for 10 min produced extinction. Conditioned rats exposed to the CS- during the 10 min recall session serve as a powerful control group because although they underwent a very similar behavioural experience to the CS+ re-exposed group they do not show extinction irrespective of the behaviour within the extinction trial. The EXT animals used in the EXTNOR microarray experiment re-exposed to the conditioned context (CS+) showed a reduction in conditioned freezing behaviour between the first two and last two minutes of the 10 min recall session indicating within-session extinction (Fig 2B). Within-session extinction is often correlated with, although not necessary for, between-session extinction indicative of successful extinction encoding [47]. There was no difference between the conditioned response to the context between the first and last two minutes of the recall session for the NOR group exposed to the CS-. Thus, only the EXT group showed within-session extinction (S2 Fig). In the rats prepared for the follow-up qPCR, immunoblotting and ChIP-qPCR studies (Fig 2C and 2D), there were no between-group differences in freezing behaviour during CFC or the first two minutes of recall (Fig 2D). Similar to the animals in the EXTNOR array experiment, there was a significant decrease in freezing responses between the first and last two minutes of the 10 min recall session indicating within-session extinction of the conditioned response.

Note that the levels of pre- and post-shock freezing and conditioned fear during the first 2 min of the recall for the extinction CS+ group were not different from the trained control animals used to generate CONSOL and REC datasets [36]. This indicates that using identical conditioning procedures, the behaviour of the control rats in different experiments used to generate the EXTNOR, CONSOL and REC datasets was independent of batch and operator across time.

### Microarray and enrichment analysis of genes regulated after CFM extinction, retrieval and acquisition

The data discussed in this publication have been deposited in NCBI's Gene Expression Omnibus [48] and are accessible through GEO Series accession number GSE66153 (http://www.ncbi.nlm.nih.gov/geo/query/acc.cgi?acc=GSE66153)

**Fig 3:**
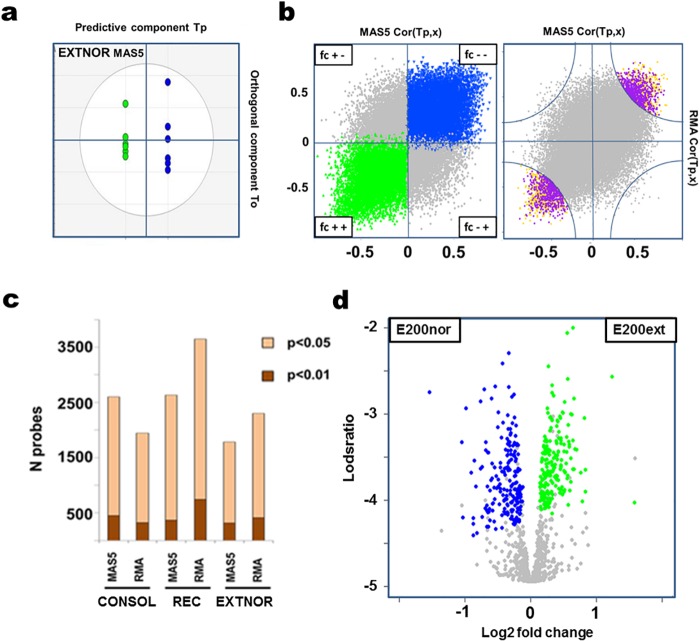
Genes identified with the extinction EXTNOR Affymetrix microarray dataset as being differentially regulated by both MAS5 and RMA normalization procedures. **a)** OPLS-DA separation of the MAS5 normalized EXTNOR data along the predictive component (T_P_, between-group variation) and the first orthogonal component (T_0_, within-group separation). EXT group: green colour, NOR group: blue colour. **b)** Scatter plots of MAS5 (x-axis) and RMA (y-axis) derived OPLS-DA loadings for each EXTNOR probe scaled as correlation coefficients Cor(T_P_, x) between -1 and 1 for the predictive component in each model. Probes in the upper right and lower left quadrants exhibit the same fold change trends, with the blue probes (left scatterplot, fc—) being down regulated and the green probes (left scatterplot fc ++) more highly expressed in in the extinction (EXT) animals. Probes (n = 969, E969) at the tips of the cloud (right scatterplot) were selected (yellow dots) when being within a radius of r = 0.75 using the outer edges as the origo and a shared VIP > 1.0, lower confidence level > 0.0 (purple dots). **c**) Bar plot showing the number of probes in each full dataset according to normalization and non-adjusted moderated t-test p < 0.05 and p < 0.01. d) Volcano plot of log2 fold change against lodsratio for of MAS5 normalized E969 candidate set with the top ranked 200 probes more highly expressed shown for each experimental group (E200ext–green, E200nor–blue).

Table 2 describes the expression datasets generated by the microarray analysis from the experimental groups. The extinction EXTNOR microarray data set was analysed using separate RMA and MAS5 normalization procedures. The previously reported CONSOL and REC datasets [36] were re-analysed using the same procedure (*data not shown*). In order to select those probes that behaved in relatively normalization-independent manner (i.e. *those genes identified as differentially regulated by both normalization procedures*), we used a supervised multivariate approach (OPLS-DA) together with jack-knifing cross-validation (Fig 3A and 3B). Univariate analysis using proportional p-value ranking showed no major differences in the numbers of differentially regulated probes between the different datasets and the normalisation procedure applied (Fig 3C). The RMA-derived data in REC had an apparently higher proportion of top-ranked genes, but this difference was not significant after OPLS-DA. This provided us with a set of differentially regulated genes for the pair-wise comparison in each dataset, and resulted in three candidate gene sets (S1–S3 Tables) of approximately the same size: C1150 (CONSOL-BDNFASO versus CONSOL-BDNFMSO, n = 1150 probes), R964 (REC-ZIFASO versus REC-ZIFMSO, n = 964 probes) and E969 (EXT versus the no recall NOR group, n = 969 probes). Again, note that the C1150 and R964 candidate gene constitute lists that are enriched for genes regulated by BDNF after CFC and Zif268 after recall, respectively.

**Table 2:** Expression data sets generated by microarray analysis from the different experimental groups.

		Within dataset analysis			Between data set analysis (CRE1016)
Memory process	Experimental group	Gene set	Candidate list after dual normalization and OPLS-DA	Top 200 upregulated candidates	CASO
CFC	BDNF-ASO	CONSOL	C1150	C200aso	CMSO
	BDNF-MSO			C200mso	RASO
Retrieval 2min Recall	Zif268-ASO	REC	R964	R200aso	RMSO
	Zif268-MSO			R300mso	EXT
Extinction	No Recall	EXTNOR	E969	E200nor	NOR
	10min Recall			E200ext	CASO

The numbers indicate number of individual genes associated with each data set.

The E969 set had 484 and 485 probes defining the no recall NOR group and EXT groups, respectively (Fig 3D). In order to increase the resolution of the functional annotation and allow for more consistent comparison between datasets, the three candidate datasets were divided into equally sized (n = 200) gene subsets according to fold change directionality. Using the sum of the moderated t-test p-values (MAS5 + RMA) as a basis for ranking, the top 200 probes more highly expressed in EXT (E200ext) and more highly expressed in NOR (E200nor) were selected for enrichment analysis (S4 Table). To reduce the likelihood of false positives in the functional association, a kappa-statistics approach was used to cluster enriched ontology terms according to semantic similarity and gene content (term significance EASE p<0.01, kappa score: 0.70, minimum terms per cluster = 3, and minimum genes per term = 5 genes). The more highly expressed genes in the extinction group (E200ext) demonstrated a clear clustering of enrichment terms (12 clusters encompassing 52 GO terms and 4 Panther terms) centred around a set of 120 genes (Table 3), whereas no similar clear clustering of annotation ontology terms was seen for genes down-regulated in extinction (i.e. those genes more highly expressed in NOR). Furthermore, reducing or increasing the top ranked number of genes or increasing the EASE cut-off to p<0.05 did not provide any clear clusters of genes/ontology terms. Finally, combining E200ext and E200nor into one enrichment analysis gave results very similar to E200ext proving the specificity of the EXT-specific functional clusters (*data not shown*).

**Table 3:** Within-experiment enrichment analysis.

Data	CONSOL		REC		EXTNOR	
Expression set	C200mso	C200aso	R200mso	R200aso	E200ext	E200nor
Memory state	Consolidation	Impaired consolidation	“Reconsolidation”	Impaired “reconsolidation”	Extinction	No extinction
**CA1 expression**	More highly expressed	More highly expressed	More highly expressed	More highly expressed	More highly expressed	More highly expressed
**Combined genes**^**1**^	64	9	93	0	120	0
**Ontology clusters**^**2**^	*Organismal development (5T*, *51G)*,	*Synaptic transmission (4T*, *9G)*	*Response to bacterium (6T*, *17G)*	*NS*	*Organismal development (6T*, *66G)*	*NS*
	*Carboxylic acid metabolism (4T*, *16G)*		*Response to stress (4T*, *14G)*		*Regulation of developmental process (3T*, *32G)*	
			*Inflammatory response (3T*, *35G)*		*Positive regulation of cell development (4T*, *20G)*	
			*Positive immune system regulation (5T*, *20G)*		*Regulation of cell motion (4T*, *10G)*	
			*Immune system development (3T*, *13G)*		*Vasculature development (3T*, *15G)*	
			*Immune-cell motion (13T*, *27G)*		*Tube morphogenesis (11T*, *17G)*	
			*Positive regulation of metabolic process (3T*, *36G)*		*Cell adhesion (3T*, *14G)*	
			*Signal transducer activity (3T*, *31G)*		*Hormone response (5T*, *36G)*	
			*Peptidase inhibitor activity (3T*, *9G)*		*Nutrient response (3T*, *14G)*	
			*Cytokine activity (3T*, *10G)*		*Plasma membrane (5T*, *77G)*	
					*Signal transducer activity (5T*, *41G)*	
					*Tyrosine protein kinase receptor (5T*, *13G)*	

Clusters of enriched ontology terms in the 200 probes for six expression sets (C200mso, C200aso, R200mso, R200aso E200ext and E200nor), representing the top 200 up-regulated and down-regulated expressed genes in the Affymetrix datasets associated with consolidation (CONSOL; up-regulated, C200mso and down-regulated, C200aso), recall (REC; up-regulated, R200mso and down-regulated, R200aso) and extinction (EXTNOR; (up-regulated, E200ext and down-regulated, E200nor).

^1^ Combined number of genes with unique identities.

^2^ Enrichment analysis using the array as reference (EASE < 0.01, kappa threshold = 0.7). Cluster classifications are followed by number of ontology terms in cluster and number of unique genes (T, G). NS is non-significant for cut-offs.

The combined 120 genes up-regulated in extinction (S5 Table) identified in the enrichment analysis were associated with different aspects of developmental and vascular processes (five clusters including organismal and vascular development, tube morphogenesis, regulation of developmental process and cell development), cell motility, cell adhesion, stimuli responses (hormone and nutrient hormone response) and plasma membrane and signal transduction (in particular tyrosine protein kinase receptor activity). The developmental and vascular associations were partly dependent on the presence of differentially expressed TGFβ and PDGF family genes and the chemokine Cxcl12 (Table 4, for annotation and distribution of candidate genes in the KEGG reference pathway for cytokine-cytokine receptor interaction). An additional analysis of protein domain structure found that a subset of the developmental genes (10 genes: *Csfr1*, *F11r*, *Fn1*, *Igf1r*, *Il6st*, *Kirrel3*, *Lsr*, *Ntrk2*, *Ptprfb*, *Ptprfd*) either belonged to tyrosine protein kinase receptors, and/or possessed immunoglobulin-like domains and/or a fibronectin-domain in addition to being involved with either the regulation of cell communication and/or differentiation.

**Table 4:** Gene candidates in KEGG reference cytokine-cytokine receptor interaction pathway after within-experiment enrichment analysis.

Classification	CONSOL	REC	EXTNOR	OPLS-DA-set	Expression Set	k-means*
Chemokines		***Cxcl1***		R964	R200mso	Profile CD
		***Cxcl3***		R964	R200mso	
		***Cxcl9***		R964		Profile CD
		***Cxcl10***		R964		
		***Cxcl11***		R964		Profile CD
		***Cxcl13***		R964	R200mso	
		***Cxcr2***		R964	R200mso	Profile CD
			***Cxcl12***	E969	E200ext	
CC-subfamily		***Ccl2***		R964		
		***Ccl3***		R964	R200mso	Profile CD
		***Ccl4***		R964	R200mso	
		***Ccl6***		R964	R200mso	
		***Ccl9***		R964		
		***Ccl20***		R964		Profile CD
		*Ccl27*		R964	R200aso	
			*Ccbp2*	E969	E200nor	
Hematopoietins	*Epor*			C1150		
	***Il11ra1***			C1150	C200mso	
	***Prlr***			C1150		
		***Csf2ra***		R964		
		***Csf3r***		R964		
		***Il6***		R964	R200mso	
		***Il13ra1***		R964		Profile CD
			***Il6st***	E969	E200ext	
			*Il23a*	E969	E200nor	
PDGF-family	***Pdgfra***			C1150	C200mso	Profile A
		***Csf1***		R964	R200mso	Profile CD
		***Kitlg***		R964		
		*Vegfc*		R964		
			***Csf1r***	E969	E200ext	
			*Egfr*	E969		
			***Flt1***	E969	E200ext	
			***Kdr***	E969		
			***Pdgfd***	E969		
			*Pdgfrb*	E969		
Interferons			***Ifnar1***	E969	E200ext	Profile A
Il10-family		***Il10rb***	***Il10rb***	R964, E969	E200ext	
TNF-family	***Tnfsf13***			C1150	C200mso	Profile B
		***Tnfrsf1a***		R964		
		***Tnfrsf1b***		R964	R200mso	
		***Tnfrsf12a***		R964	R200mso	Profile CD
		***Tnfrsf14***		R964		Profile CD
		***Tnfrsf26***		R964	R200mso	
			*Tnfrsf4*	E969		
			***Tnfrsf11b***	E969	E200ext	
			*Tnfrsf14*	E969	E200nor	Profile CD
			***Tnfsf12***	E969		Profile A
TGFβ -family	***Bmp4***			C1150	C200mso	Profile B
	***Tgfaa***			C1150	C200mso	
			***Bmpr2***	E969	E200ext	
			***Tgfb2***	E969		
			***Tgfb3***	E969		
			***Tgfbr2***	E969		Profile A
Il1-family		***Il1a***		R964	R200mso	Profile CD
		***Il1b***		R964		
		***Il1rn***		R964		

Genes more highly expressed in the groups that are associated with consolidation, recall and extinction (CMSO, RMSO and EXT, respectively) are in **bold**. Genes down regulated in those states that are not in bold.

**Fig 4:**
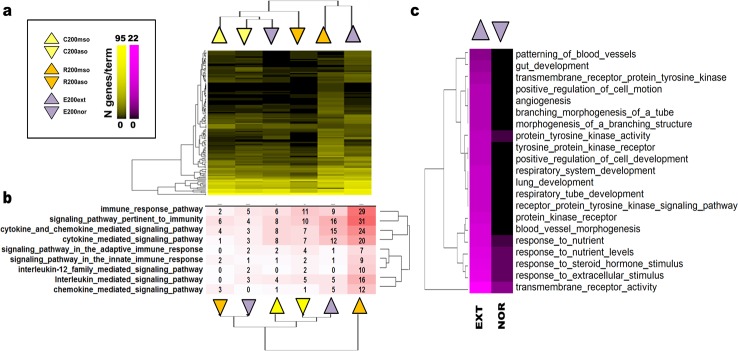
Within-experiment enrichment analysis shows that extinction and recall were associated with distinct immunity-related and cytokine-and chemokine-mediated signalling pathways. Complete linkage clustering of genes present in the enriched ontology terms in the six different expression-sets representing the top 200 up-regulated and down-regulated for consolidation (C200mso, C200aso), recall (R200mso, R200aso) and extinction (E200ext, and E200nor) for **a)** GO and Panther ontologies showing that gene networks regulated during extinction and recall are more similar to each other than those regulated in consolidation. **b)** In the same datasets, Pathway (PW) ontology shows that recall and extinction are both associated with the regulation of immunity-related genes. E200ext had four times the enrichment of genes associated with these pathways than E200nor, but R200mso had twice as many as E200ext **c)** Comparison between E200ext and E200nor for a number of genes present in GO and Panther ontology terms where the E200ext annotation has > 4 fold more genes than corresponding E200nor annotation or where the E200nor annotation has 0 genes. Yellow highlight = GO/Panther ontologies enriched in all expression sets. Pink highlight = GO/Panther ontologies enriched in EXTNOR expression sets.

* k-means clustering for the pattern or profile of fold changes across all data sets (see Fig 4).

For a systematic comparison to the E200ext and E200nor gene sets, the top and bottom 200 genes from the C1150 (C200mso, C200aso, S6 Table) and R964 (R200mso, R200aso, S7 Table) candidate sets were selected and analysed for functional associations as described above. Enrichment clusters based on ontology terms with at least 5 common genes (p < 0.01, kappa score < 0.70) were highly expressed and mainly seen in the expression sets C200mso, R200mso, and E200ext which represent the behavioural associated with the consolidation, retrieval and extinction of CFM, respectively. The C200mso and C200aso datasets contained three clusters of functionally related ontology terms and genes (Table 3). Two clusters with more highly expressed probes (64 genes combined) associated with *developmental processes* and *carboxylic acid metabolism*, and one smaller down-regulated cluster (nine genes) associated with *synaptic transmission* (S8 Table). The selected R200mso and R200aso sets had 10 clusters of enrichment terms (93 genes combined, S9 Table) associated mainly with immune-associated ontology terms, but also signal transduction (31 genes) and peptidase inhibitor activity (nine genes). When comparing genes in significantly enriched ontology terms present in the clusters (a total of n = 122 terms from the GO and Panther ontologies across the C200mso, C200aso, R200mso, R200aso, E200ext and E200nor expression sets), analysis revealed that R200mso and E200ext were somewhat more similar to each other than either of the CONSOL expression sets (C200mso and C200aso) (Fig 4A). Therefore, the gene regulatory networks engaged by the two retrieval conditions were more analogous to each other than those regulated during consolidation.

Using the Pathway ontology for annotation, a clear difference was detectable for immunity-related and cytokine-and chemokine-mediated signalling between E200ext and E200nor, with E200ext showing a four times—enrichment of genes associated with these pathways (Fig 4B). Ontology terms containing such genes were also those that were most categorically different when comparing the E200ext and E200nor gene annotations (Fig 4C). The in-depth annotation of immune associated-genes using the Pathway ontology also found that R200mso separated out from the other sets by having approximately twice as many immunity pathway-associated genes compared to E200ext and three times as many as C200mso or C200aso. In contrast, there were only a few such genes more highly expressed in R200aso (Fig 4B). The Pathway annotation of R200mso immune-associated signalling genes were dominated by genes including *Il1a*, *Il1b*, *Il6*, *Il1rn*, *Cxcl1*, *Ccl3*, *Ccl4*, *Nfkb2*, and *Nfkbia* (Table 5). The C200mso and C200aso expression sets had similar number of immune-associated genes whereas immune-associated genes were enriched in R200mso and E200ext compared to their corresponding control subsets (R200aso, E200nor). R200mso had at least twice as many immune-associated genes than E200ext, which in turn had twice as many immune-associated genes than the CONSOL subsets. Overall, these data suggest that retrieval that does not lead to extinction (2 min recall) and retrieval that initiates extinction of memory (10 min recall) engaged the immune system but with distinct profiles.

**Table 5:** List of probes/genes part of the ‘Regulation of immune response’ clusters associated with the consolidation (CONSOL), Recall (REC) and extinction (EXTNOR) Affymetrix datasets.

Affymetrix probe set identifier	Gene	averC_log2fc	averR_log2fc	averE_log2fc
1367614_at	*Anxa1*	0.471954214	**-1.01806075**	0.152025972
1368482_at	*Bcl2a1d*	0.615208059	**-1.3737525**	-0.031342877
1376652_at	*C1qa*	-0.198655566	-0.402882	0.111350596
1368000_at	*C3*	-0.385276765	**-1.054185**	0.099718505
1369815_at	*Ccl3*	0.992833381	**-1.6063375**	-0.052842573
1387952_a_at	*Cd44*	0.057897444	**-0.56865975**	-0.162831776
1389470_at	*Cfb*	0.351180468	-0.53460425	**0.429031305**
1379631_at	*Csf1*	0.13713089	**-0.24522075**	-0.031237887
1373544_at	*Cxcl9*	0.510267702	-0.6449735	0.133538639
1373575_at	*FCER1G*	-0.038082937	-0.52119225	0.09898661
1367850_at	*Fcgr2a*	0.228680455	**-0.76906975**	0.025279774
1387378_at	*Fcnb*	0.556963045	-1.13802325	-0,111652691
1368870_at	*Id2*	-0.113241961	**-0.3939355**	-0.113350171
1388711_at	*Il13ra1*	0.098656069	**-0.4285345**	0.109734882
1371170_a_at	*Il1a*	1.071548576	**-1.1511425**	0.154106745
1368592_at	*Il1a*	1.057774384	**-1.145035**	-0.118245924
1389528_s_at	*Jun*	0.230980764	-0.24443225	0.186544707
1369788_s_at	*Jun*	0.012255876	-0.196955	0.146698163
1382346_at	*LYN*	-0.07484352	-0.402691	**0.341284111**
1398275_at	*Mmp9*	**0.342944888**	-0.182634	0.492303745
1374468_at	*Myd88*	0.145706079	-0.35968925	0,100339349
1389538_at	*Nfkbia*	0.366094429	**-0.66984925**	0.272595961
1368939_a_at	*Ntrk3*	0.030353182	**-0.24740425**	0.026945362
1371250_at	*Pf4*	0.044128028	**-0.33221375**	0.144107957
1370177_at	*PVR*	0.586055518	**-0.5282015**	-0.009308002
1371213_at	*RT1-A3*	**0.328839252**	-0.128917	-0.09465224
1392107_at	*Sbno2*	0.141481708	**-0.5787715**	0.079482048
1372254_at	*Serping1*	-0.153994136	-0.742713	0.030125108
1369584_at	*Socs3*	0.309677493	-0.58882225	-0.22957273
1369943_at	*Tgm2*	0.059985502	**-0.56095425**	-0.22627132
1376327_at	*Tnfrsf14*	0.274691802	-0.938031	-0.103727588
1388130_at	*Zyx*	0.167608943	**-0.32025875**	-0.021894281

Average of MAS5 and RMA derived Log2 fold change for CONSOL comparison (averC_log2fc: negative CMSO, positive CASO), REC comparison (averR_log2fc: negative RMSO, positive RASO), and EXTNOR comparison (averE_log2fc: negative NOR, positive EXT). Statistical significance ranking indicated by the sum of both MAS5 and RMA derived moderate p-values (sump < 0.1 = **bold**, sump < 0.2 = underlined). The Gene column is sorted in alphabetical order.

These data suggests that extinction is correlated with the regulation of genes associated with development, hormonal and nutrient responses and cell adhesion/communication, with the regulation of tyrosine protein kinase-, cytokine- and chemokine-signalling all significantly affected. In addition, similar to 2 min retrieval, extinction is strongly associated with changes in immunity-related and cytokine-and chemokine-mediated signalling.

### Comparison of the transcriptomes associated with extinction, retrieval and consolidation

**Fig 5:**
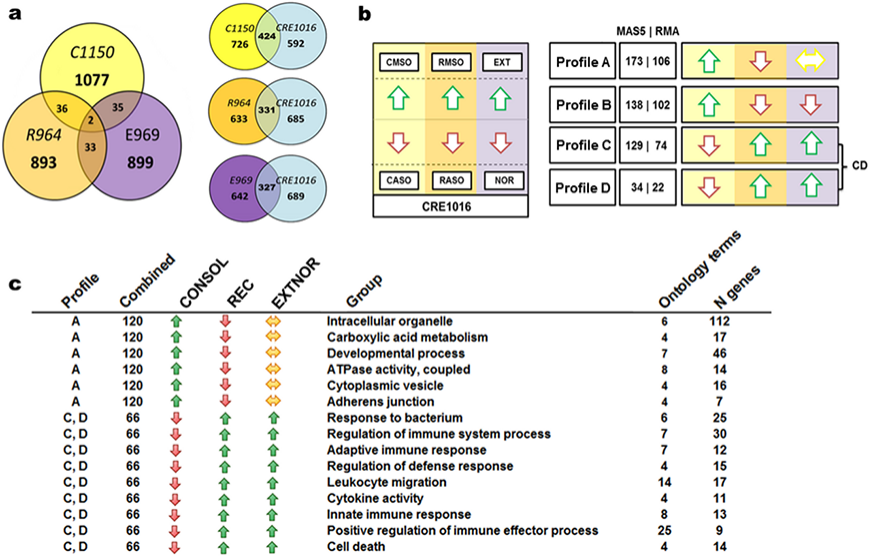
Differential gene regulation profiles in CA1 associated with the consolidation, recall and extinction of CFM. **a)** Venn diagram for overlap between OPLS-DA derived candidate datasets (C1150, R964, and E969). A total of 1016 probes (CRE1016) are part of at least one candidate set while having a normalization independent up- and down regulation behaviour within the separate experiments. **b)** k-means clustering of MAS5 and RMA log2 fold change profiles for the CRE1016 probes found four clusters (A, B, C, D) to be similar between normalizations in their silhouettes and gene content (number of genes shown in brackets as MAS5 | RMA). Coloured arrows show in which experimental group the cluster genes are more highly expressed (CASO, RASO, NOR: red arrow; CMSO, RMSO, EXT: green arrow). The fold change for the EXT vs. NOR comparison was very weak (log2 fold change |< 0.10|) in both MAS5 and RMA data and was classified as being unchanged (yellow horizontal arrow). **c)** The table shows the groups of significantly enriched ontology clusters (EASE < 0.01, kappa 0.7) present in the different fold change profiles (cluster A with 120 genes & fused clusters C and D with 66 genes) with the number of ontology terms and genes they encompass. Cluster B did not result in any significantly enriched ontology clusters. The ontology enrichment analysis is based on annotations provided by Gene ontologies (GOBP, GOCC, and GOMF) and Panther ontologies (Biological process, Molecular function).

The distinct nature of the different gene expression patterns was indicated by very little overlap of individual top-ranked gene candidates’ overlap between the different candidate sets E969, R964 and C1150 (Fig 5A). In order to investigate if there are subsets among candidates that were likely to be co-regulated and influenced by more than one condition, the top OPLS-DA derived candidates were selected (CRE1016, total of n = 1016 probes, S10 Table) and both their MAS5 and RMA derived log2 fold-change used for k-means clustering. The combined candidate set was divided into 6 fold-change-dependent profile clusters (k-means clustering) of which four were observed in the fold-change patterns between MAS5 and RMA sets (Fig 5B, S11 Table). The fold-change profiles were strongly defined by having a reversed relation between the control ASO and the experimental MSO groups in the CONSOL and REC gene sets. Genes within profile A (120 combined genes) were more highly expressed in CMSO, down-regulated in RMSO and very weakly regulated in EXTNOR (|0 < log2 fold change < 0.2|, designated as unchanged in Fig 5C). Thus, profile A genes represents those whose expression have a trend towards being was up-regulated during consolidation and down-regulated following 2min recall that does not lead to extinction. Profile A had an enrichment of genes associated with the same *organismal development* and *carboxylic acid metabolism* previously identified in the C200mso expression set (Fig 5C). Additional sets of overrepresented genes with profile A were classed as having the molecular function of *ATPase activity* and the cellular compartment localization of *organelles/vesicles* and *adherence junctions*. The ATPase activity was related to cellular ion homeostasis and transport. The organelles involved were mainly mitochondria, lysosomes and the endoplasmatic reticulum. Profile B did not provide a clear enrichment pattern. Due to their similar expression patterns, profiles C and D where fused into one set (66 combined genes) and was characterised by genes whose expression had a trend towards being down-regulated in CMSO, up-regulated in RMSO and somewhat weakly in EXT. This profile corresponds to genes down regulated during consolidation and up regulated following recall initiated by conditioned context exposure for either 2 min or 10 min. This profile was characterised by the immune-associated genes/ontology terms previously seen in the R200mso group. Based on the total number of annotated cytokines regulated in the CRE1016 set, chemokines and beta-chemokine receptor ligands together with tumour necrosis factor (TNF)-family genes defined the reconsolidation-associated RMSO group, whereas TGFβ and PDGF family genes were more characteristic for the EXT group (see Table 4). The only chemokine exception for EXT was Cxcl12. It should be noted that the profile patterns in Fig 5B only indicate that differential regulation of gene expression was measured in the CONSOL, REC and EXTNOR experiments and the valancy of the changes. It does not allow any conclusion about the magnitude of the change in expression of individual genes between the experiments.

### Validation of candidate gene expression with retrieval using qPCR

**Fig 6:**
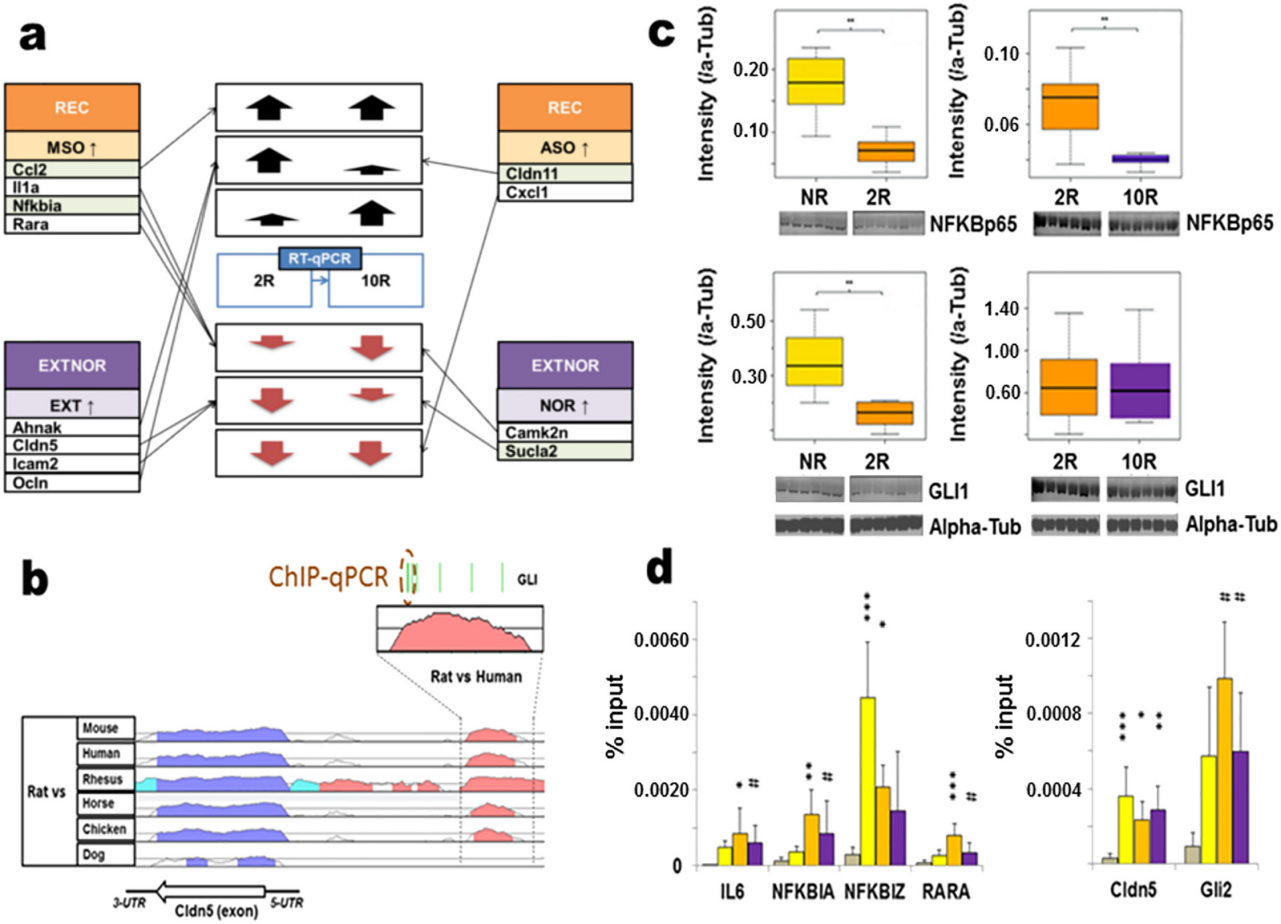
Validation of gene expression and promoter analysis associated with recall and extinction. **a)** RT-qPCR results for a set of REC and EXTNOR dataset candidates. All genes with p-values p < 0.15 (Dunnett’s test, REC and EXTNOR groups against No recall group) are shown (light green coloured genes have a 0.10 < p < 0.15). **b)** Comparative genomics overview of the predicted GLI1 binding site investigated by ChIP-qPCR shows the *Cldn5* gene in the Rat genome as compared to the genomes of Mouse, Human, Rhesus macaque, Horse, Chicken and Dog and where colour signifies >80% sequence similarity. Green stripes show the different conserved sites of predicted GLI1 binding between Rat and Human. **c)** Comparison Gli1 and RelA (NF-κBp65) protein levels in CA1 between No recall (yellow boxes, n = 6) and 2min recall (orange boxes, n = 6) and 2min recall and 10min recall (purple boxes, n = 6) using background and alpha-tubulin intensity for normalization. Pair-wise comparison of expression differences within gel runs was conducted by Welsh’s t-test. Data are represented as box-and-whisker plots of the median and interquartile ranges. **d)** *Left panel*; ChIP-qPCR of predicted binding sites for NF-κBp65 against promoter elements upstream of *Il6* (overlapping with the first exon), *Nfkbia* (overlapping with the first exon), *Nfkbiz* (overlapping with the first exon), *Rara* (2200 bases upstream of the first aligned mouse exon). *Right panel*; ChIP-qPCR of predicted binding sites for GLI1 against promoter elements for *Cldn5* (720 upstream of the first exon) and *Gli2* (300 bases upstream of the first exon) respectively. Dunnett’s test based significance in enrichment of No recall (yellow bar, n = 6), 2min recall (orange bar, n = 6) and 10min recall (purple bar, n = 6) against negative control (IgG, tanned bar, n = 6) is shown. Data are shown as Mean +/- SD. *** p < 0.001, ** p < 0.01, * p < 0.05, # p < 0.10.

Several candidate genes with annotations covering the range of the GO annotations were selected from the REC and EXTNOR top candidates for qPCR analysis (Table 6). The expression of the following genes were analysed from REC: *Il1a*, *Cxcl1*, *Ccl2*, *Cldn5*, *Nfkbia*, *Rara*. A number of genes present in the E200ext (*Ahnak*, *Cldn5*, *Icam2*, *Ocln*) and E200nor (*Camk2n1*, *Cldn11 Sucla2*, *Egr3*) were also analysed by qPCR. Overall, the expression of nine genes out of the 13 investigated had a p-value <0.05 (*Camk2n1*, *Cldn5*, *Il1a*, *Cxcl1*, *Icam2*, *Rara;* p<0.05) or near that threshold (*Ocln*, *Ahnak*, *Cxcl1*, p<0.10) in a comparison to the No recall (NR) group (Fig 6A). The remainder of tested candidate genes (*Ccl2*, *Cldn11*, *Nfkbia*, *Sucla2*) had p<0.15 and one (*Egr3*) was at p>0.3 (data not shown). The expression of *Il1a* was previously found to be up-regulated after 2 min recall in the CA1 [36]. *Opalin*, a gene with a lower RMA p-values (CONSOL p = 0.028; REC p = 0.056; EXTNOR p = 0.058) and higher MAS5 p-values (CONSOL p = 0.314; REC p = 0.575; EXTNOR p = 0.079), was top-ranked in all three datasets but was unchanged when assessed by qPCR, indicating it to be a RMA-normalization artefact (data not shown). The fact that most genes validated with qPCR are differentially expressed either between No recall and 2 min recall or the No recall and 10 min recall groups confirms the relevance of the microarray results. Importantly, because different experimental strategies were used to measure gene regulation in extinction in the qPCR and microarray experiments (i.e. behavioral vs. ASO with behavior), the changes in gene expression we measured closely correlate with the underling post-retrieval memory processes and not to training artefacts related to the individual approaches.

**Table 6:** Regulated genes selected for RT-qPCR with their GO annotations.

Gene	GO annotation (biological process)^1^	Selection dataset	MAS & RMA dataset values^2^,^3^	qPCR data^4^ (NR vs. 2R or 10R)
*Ahnak*	GO:0007399—nervous system development	EXTNOR	MAS5: p < 0.0001, Log2fc = 0.680, VIPlow = 1.669	NR vs 2R: p = 0.078, Fc = 1.56
			RMA: p < 0.0001, Log2c = 0.514, VIPlow = 2.862	NR vs 10R: p = 0.465, Fc = 1.26
*Camk2n1*	GO:0009987—cellular process	EXTNOR	MAS: p = 0.023, Log2fc = -0.318, VIPlow = 0.740	NR vs 2R: p = 0.492, Fc = 0.930
	GO:0007154 –cell communication		RMA: p = 0.004, Log2c = -0.353, VIPlow = 1.509	NR vs 10R: p = 0.032, Fc = 0.660
	GO:0007267—cell-cell signalling			
	GO:0007268—synaptic transmission			
*Ccl2*	GO:0002376—immune system process	REC	MAS: p = 0.091, Log2fc = -1.019, VIPlow = 0.844	NR vs 2R: p = 0.150, Fc = 1.470
	GO:0006928—cell motion		RMA: p = 0.056, Log2c = -1.162, VIPlow = 1.915	NR vs 10R: p = 0.176, Fc = 1.290
	GO:0006935 –chemotaxis			
	GO:0006950—response to stress			
	GO:0050900—leukocyte migration			
*Cldn5*	GO:0007399—nervous system development	EXTNOR	MAS: p = 0.025, Log2fc = 0.532, VIPlow = 3.577	NR vs 2R: p = 0.017, Fc = 1.770
	GO:0007154—cell communication		RMA: p = 0.010, Log2c = 0.452, VIPlow = 1.128	NR vs 10R: p = 0.300, Fc = 1.390
	GO:0001508—regulation of action potential			
	GO:0007155—cell adhesion			
	GO:0042552 –myelination			
*Cldn11*	GO:0003008—system process	REC	MAS p = 0.094, Log2fc = 0.187, VIPlow = 0.051	NR vs 2R: p = 0.114, Fc = 1.200
	GO:0007399—nervous system development		RMA: p = 0.090, Log2c = 0.164, VIPlow = 0.355	NR vs 10R: p = 0.447, Fc = 0.950
	GO:0007154—cell communication			
	GO:0001508—regulation of action potential			
	GO:0007155—cell adhesion			
*Cxcl1*	GO:0006950—response to stress	REC	MAS: p = 0.029, Log2fc = 1.100, VIPlow = 1.289	NR vs 2R: p = 0.055, Fc = 0.490
	GO:0002376—immune system process		RMA: p = 0.042, Log2c = 1.078, VIPlow = 1.221	NR vs 10R: p = 0.066, Fc = 0.500
	GO:0006935 –chemotaxis			
	GO:0016477 –cell migration			
	GO:0050900—leukocyte migration			
*Il1a*	GO:0006950—response to stress	REC	MAS: p = 0.023, Log2fc = -1.215, VIPlow = 1.160	NR vs 2R: p = 0.700, Fc = 0.980
	GO:0002376—immune system process		RMA: p = 0.035, Log2c = -1.122, VIPlow = 1.874	NR vs 10R: p = 0.014, Fc = 0.490
	GO:0007165—signal transduction			
	GO:0043122—regulation of IKB kinase/ NF-κB			
*Icam2*	GO:0007155—cell adhesion	EXTNOR	MAS: p = 0.003, Log2fc = 0.485, VIPlow = 0.872	NR vs 2R: p = 0.095, Fc = 0.690
	GO:0009987—cellular process		RMA: p = 0.018, Log2c = 0.393, VIPlow = 0.769	NR vs 10R: p = 0.144, Fc = 0.740
	GO:0007155—cell adhesion			
*Nfkbia*	GO:0002376—immune system process	REC	MAS: p = 0.018, Log2fc = -0.683, VIPlow = 1.143	NR vs 2R: p = 0.152, Fc = 1.110
	GO:0002224—toll-like receptor signaling		RMA: p = 0.020, Log2c = -0.665, VIPlow = 1.913	NR vs 10R: p = 0.390, Fc = 0.790
	GO:0032088—negative regulation of NFKB			
*Ocln*	GO:0045216—cell-cell junction organization	EXTNOR	MAS: p = 0.017, Log2fc = 0.406, VIPlow = 0.314	NR vs 2R: p = 0.012, Fc = 1.450
	GO:0046500—S-adenosylmethionine metabolic process		RMA: p = 0.006, Log2c = 0.428, VIPlow = 0.978	NR vs 10R: p = 0.133, Fc = 1.260
*Rara*	GO:0021766—hippocampus development	REC	MAS: p = 0.014, Log2fc = -1.210, VIPlow = 1.879	NR vs 2R: p = 0.693, Fc = 0.95
	GO:0048167—regulation of synaptic plasticity		RMA: p = 0.007, Log2c = -0.175, VIPlow = 0.978	NR vs 10R: p = 0.025, Fc = 0.490
	GO:0031641—regulation of myelination			
	GO:0006950—response to stress,GO:0007165—signal transduction			
*Sucla2*	GO:0008152—metabolic process	EXTNOR	MAS: p = 0.092, Log2fc = -0.288, VIPlow = 0.146	NR vs 2R: p = 0.118, Fc = 1.24
	GO:0006099—tricarboxylic acid cycle		RMA: p = 0.020, Log2c = -0.131, VIPlow = 1.118	NR vs 10R: p = 0.734, Fc = 0.970
	GO:0006105—succinate metabolic process			

1 Representative gene ontology biological processes (GOBP) annotations.

2 Median log2 expression of gene in specific dataset and over all three datasets (CONSOL + RECON + EXTNOR).

3 Moderated t-test p-values from Affymetrix datasets.

4 Dunnett’s test (No recall “0R” vs. 2 min recall “2R” or No recall “0R” vs 10 min recall “10R”). Normalized against housekeeping genes (*Gapdh*, *Yhwaz*).

### Promoter analysis of genes regulated upon retrieval

The distinctive nature of the gene expression patterns made it feasible to investigate the gene regulatory network structure associated with consolidation, recall and extinction. In order to investigate the mechanisms leading to altered gene expression associated with extinction, we first used phylogenetic footprinting analysis to predict evolutionary conserved transcription factor binding in the gene sets identified with the retrieval and extinction of CFM in the enriched ontology term clusters R200mso (94 genes) and E200ext (120 genes), respectively. Orthologous genes between rat, mouse and human were analysed approximately 2000 bases upstream of their transcription start sites (TSS). The top five transcription factor candidates from this analysis were NF-κB and IK1 (associated with the genes of the R200mso dataset) and AP4, Egr-1, GLI1 (associated with the E200ext dataset, data for GLI sites upstream of the TSS of orthologous *Cldn5* is shown as an example in Fig 6B). Two transcription factors, NF-κBp65 (also known as RelA) and GLI1, were then selected to directly measure their activities after recall. NF-κBp65 was chosen because NF-κB signalling has recently been implicated in regulating post-retrieval memory processes [37, 38, 49]. GLI was chosen because binding sites for transcription family members GLI1, GLI 2 and GLI3 were enriched in the 86 genes cluster for multicellular development associated with extinction therefore GLI may represent a novel mechanism controlling transcriptional events required for extinction.

qPCR analysis revealed no differences in NF-κBp65, GLI1, GLI 2 and GLI3 mRNA levels in the CA1 of the control *No recall*, *2min recall* and *10min recall* groups (data not shown). In contrast, using the same CA1 samples Western blotting showed that the protein levels of NF-κB p65 and GLI1 were altered after recall (Fig 6C). NF-κBp65 protein was down regulated after a 2 min conditioned context exposure and was further reduced if the rats had a prolonged 10 min recall trial. Protein levels of GLI1 were down regulated to the same extent under both recall conditions.

ChIP-qPCR analysis of NF-κBp65 binding on four candidate genes (*Il6*, *Nfkbia*, *Nfkbiz*, *Rara*) demonstrated NF-κBp65 binding to *Il6*, and *Nfkbia* after 2 min recall and to *Rara* after 10 min recall (Fig 6D). NF-κBp65 was bound to the *Nfkbiz* gene in the *No recall* control but not after recall. GLI1 binding to predicted sites in the *Cldn5* promoter region within 2000 bases of TSS was demonstrated but there were no change in the GLI1-*Cldn5* interaction with the recall of CFM (Fig 6D). There was a trend (p<0.10) for the binding of GLI to elements in the *Gli2* promoter after recall. Binding of GLI1 to a predicted GLI1 binding site upstream of the *Six4* gene was not observed (data not shown).

Thus we showed that NF-κB activity in the CA1 was regulated after the recall of CFM and furthermore we showed that there was the differential binding of this transcription factor to identified genes with recall conditions not associated with extinction and extinction of the memory.

## Discussion

To gain insight into the molecular signatures of memory processes, we used a systematic analytic approach exploiting microarray data to investigate the earliest regulation of the secondary gene expression in the CA1 of the hippocampus associated with the extinction of CFM in rats, and compared this pattern of regulation to the profiles obtained within the same region at the same times after conditioning and retrieval. The two-hour time point chosen for analysis represents the genes that are likely to be directly regulated by transcription factor activity initiated within 30 minutes after memory acquisition and recall [38, 50]. Our investigation of the gene networks in the CA1 following consolidation the extinction of CFM revealed marked differences in the regulation neuroimmunological gene expression when compared to consolidation and the retrieval of CFM in the absence of extinction.

We found very little gene overlap between the top-ranked 1000 genes regulated in the separate memory processes. This indicated that extinction has a distinct molecular substrate. The 200 top ranked up-regulated genes in extinction were dominated by those associated with developmental processes (organismal, cellular and vascular), cell adhesion, hormone responses, nutrient hormone responses and signal transduction pathways, particularly those involving TNF and TGFβ, and PDGF tyrosine-kinase receptor activity. An analysis of the protein domains in extinction-regulated genes inherent to the developmental-associated processes found that they contained immunoglobulin-like domains that represented a subset of beta-cytokines/cytokine receptors and/or possessed fibronectin-domains, and were involved in regulation of cell communication and/or differentiation.

We observed that a subset of immune-response genes fitted the profile of being down regulated during consolidation and up regulated after the recall of CFM. However, there was a distinction between the immune-response genes regulated by the different conditions of recall. The immunity-associated signalling ontologies associated with recall of 2 min duration were dominated by proinflammatory response genes, such as IL-1, IL-6, chemokines, beta-chemokine receptor ligands and tumour necrosis factor (TNF)-family genes. This profile of regulated functions was seen in an earlier analysis of the REC dataset [36]. Prolonged recall leading to extinction was however characterized by immunity–associated genes associated with the TGFβ, PDGF and TNF-family genes. While cytokines are commonly associated with cell-to-cell communication in the immune system, they are expressed in normal adult brain and play an active role in cellular events that induce structural changes at the synaptic level [51, 52].

TNFα is one of the most potent mediators of AMPA receptor (AMPAR) trafficking inherent to the regulation of synaptic strength [53]. AMPAR trafficking plays role in both reconsolidation-like processes and extinction [54, 55]. In particular, transient waves of reductions and increases in surface AMPAR are prerequisite for the destabilization processes that underlie the memory strengthening and updating functions ascribed to reconsolidation [56]. The regulation of TNF family genes with recall provides evidence of a mechanistic link between TNFα-signalling events driving AMPAR insertion into synapse and the adaptive memory reorganization characteristics described for both reconsolidation-like processes and extinction [16, 57–59]. Extinction was additionally characterized by TGFβ and PDGF signalling events. There is currently no direct evidence that TGFβ and PDGF modulate post-retrieval memory processes. Members of the transforming growth factor-β (TGFβ) family are however involved in the modulation of both excitatory and inhibitory synaptic transmission and plasticity [60–63]. LTP and hippocampal–dependent learning was disturbed in the PDGFR-β KO mice [64, 65]. The present results suggest that as significant modulators of synaptic plasticity, the activity of TGFβ and *PDGFR-β* may be relevant to extinction.

Our data support growing evidence for NF-κB signalling following retrieval in the subsequent expression of learnt responses [21, 37, 38, 66]. In addition, we identified a number of the target genes regulated by NF-κB in this process. Levels of NF-κBp65 in the CA1 were decreased two hours after recall of CFM, and this decrease was correlated with the length of the recall. This reduction may indicate the altered availability of cellular NF-κBp65 to control gene expression. Phylogenetic footprint analysis indicated that short retrieval trials led to the preferential regulation of genes with containing NF-κB and related Iκ1 sites in proximal promoter regions. The binding of NF-κBp65 to all of four candidates genes, *Il6*, *Nfkbia*, *Nfkbiz*, *Rara* was similarly regulated by the duration of recall, tightly linking DNA binding to activity. The enriched regulation of cytokines, chemokines, adhesion molecules, acute phase proteins, and inducible effector enzymes that are involved in the innate immune response we observed after short recall is perhaps not surprising since NF-κB is well known to up-regulate the expression of these classes of molecules [67]. The identification here of the regulation of NF-κB activity and the large-scale changes in the expression of NF-κB regulated genes after memory retrieval here provides compelling evidence that our experimental approach not only confirmed mechanisms already associated with recall (see below), but also validates the new insights into the regulatory control that the precise conditions of recall have on the switch to extinction.

NF-κB activity after recall forms part of determinative transcriptional switch that directs memory towards extinction [38]. In detail, de le Fuente and colleagues (2011) reported that NF-κB was required for the reconsolidation CFM initiated by short duration exposure to the conditioned context, but prolonged exposure to the same context resulted in the activation phosphatase calcineurin with the concomitant effects of NF-κB deactivation, activation the transcription factor NFAT and extinction. The sensitivity of reductions in NF-κB levels with the length of the retrieval trial we saw here reflects the transition to transcriptional events that favour extinction. It has been noted that NF-κB activity prevents extinction [38]. It is an intriguing possibility that the wide-scale expression of NF-κB regulated cytokines including IL-1 and IL-6 which are refractory for memory formation [68], also functions to prevent extinction. This hypothesis is supported by recent evidence that short retrieval events result in the activation of protein synthesis-dependent processes that *constrain* extinction rather than promote reconsolidation[69].

Phylogenetic footprint analysis indicated that prolonged context exposure, which initiated extinction, was correlated with the enriched expression of genes containing AP4, Zif268 (Egr-1) and GLI1 binding elements in their promoter regions. To date very little evidence exists for a functional role for AP4 in the adult brain. AP4 functions as a transcriptional repressor of neuronal gene expression in non-neuronal tissues [70], but can also function to regulate the phenotype specification of neurons in development [71]. Zif268 activity has been shown to be essential for reconsolidation initiated by short retrieval trials [7]. However, there is evidence that Zif268 expression is increased with prolonged conditioned context exposures, and furthermore that Zif268 activity constrains the expression of extinction [72]. Thus, in the face of new learning contingencies required for extinction [16], one functional consequence of increased Zif268 activity with retrieval is to regulate the expression of molecules that are specifically required for the maintenance of the original CS-US memory. This function of Zif268 is consistent with its role in determining memory strength during conditioning [73]. The Gli1 transcription factor has so far not been implicated in memory processes. The levels of Gli1 protein in CA1 were down regulated after both short and long recall of CFM. This indicated that Gli1 may have some form of involvement in post-retrieval processes of memory, but it does not seem to possess the same sensitivity to re-exposure duration that NF-κBp65 exhibits. Gli1 activity is regulated downstream of the Smoothened (Smo) and Patched (Ptch) receptors in the Hedgehog pathway. Both receptors are present in mature rat CA1 neuronal spines and are involved in the formation of functional synapses [74, 75]. Although we didn’t see a functional consequence of the change in Gli1 expression on target gene regulation after memory recall via ChIP analysis, this may have been a consequence of target selection or indeed time after retrieval of the assay. Nevertheless, it must be considered that the control of gene expression ultimately depends on the concerted activity of a cohort of transcriptional effectors. As such the differences in the precise configuration of transcription factor activities after memory retrieval will be the ultimate determinant of the regulation of gene expression leading to extinction.

Finally, with regard to the classification methodology employed in this study, it should be noted that all high-throughput experimental approaches tends to yield long lists of differentially expressed genes, and there is no simple or true way of deciding absolute cut-off values for inclusion–in particular with regard to probes whose intensity are close to the background noise. The use of the dual normalizations did not prove that all mono-normalization specific, highly ranked probes are devoid of biological relevance; it was merely another form of ranking employed to reduce false positives. The present focus on the classification of genes is only one example of how to move forward characterizing the gene regulatory networks underlying complex behavioural processes. As such, this particular approach was heavily dependent on gene annotation and curation of ontology databases and how to combine them with comparative genomics data. While enrichment analysis approaches are well developed, there are no coherent approaches for the use of the actual ontology terms and their more biological interpretation in a more complex context other than at the molecular or cellular level. The overrepresentation of developmental gene-associated groups in the E200ext and C200mso expression sets, was for instance, likely to a reflect of (i) the hippocampus arguably being among one of the most plastic neural regions in the adult brain and, (ii) an ontology annotation effect due to scientific fields differing in their current annotation activity and the design of molecular biology ontologies. The absence of a more diverse terminology and ontology terms for “immune-response” associated genes that reflect their more subtle neural roles (see for instance (Williamson and Bilbo 2013)) is a good example of the latter problem.

Our systematic and comparative approach to genomics data has provided an important step towards understanding the regulatory networks in CA1 underlying contextual fear memory processes. We provide a new insight for the major influence cytokine signalling has at different stages of learning and memory. This in turn is regulated by NF-κB signalling which has been shown to be refractory to extinction upon recall of memory. We suggest this may point to targeting cytokine signalling after recall as a therapeutic strategy to promote extinction mechanisms that are deficient across many psychiatric disorders [76].

## Supporting Information

S1 Fig Prolonged exposure to a fear conditioned context produced between session extinction. Rats were first pre-exposed for 3 days to two experimental chambers for 10 min/d. These contexts differed in a number of characteristics including size, spatial location, odor and lighting, and exposure to each chamber was separated by a minimum of 4 hours. The conditioning trial was given 24 hours later. Conditioning consisted of the rats being placed individually in one of the chambers (at the same time of day as during the pre-exposure sessions) for 3 min in a counterbalanced manner and a single scrambled foot shock (US, 0.5 mA for 2 s) was delivered at 2 min. The rats were then returned to their home cages. Forty-eight hours later, rats were either returned to the CS conditioned context (CS+, EXT group, light blue, n = 4) or to the other context (CS- no fear recall, NOR group, mid blue, n = 4) for 10 min. All rats were subsequently exposed to the CS+ for 2 min (Test) 48 hours later. Rats showed an increase in postUS freezing, however conditioned freezing behaviour was only seen in the EXT group exposed CS+ during the first two min of the 10 min non-reinforced extinction training (i.e. a difference in behaviour between the PostUS and Recall (1st 2 min) was seen for the NOR group only, P < 0.01). There was a trend towards a decrease in conditioned freezing measured in the last two min of the extinction in this group (within-session extinction which represents extinction memory acquisition[77]). The EXT group also showed less conditioned fear to the CS+ during the Test than the NOR group. Thus, exposure to the CS+ for 10 min is necessary for a persistent reduction in conditioned fear behaviour (between-session extinction). Statistical significance was tested for within-group differences (paired t-test) and between-group differences at test (Welch’s t-test). **P < 0.01.
(TIF)

S2 Fig Representation of the behavioural data shown in Fig 2 to highlight the freezing behaviour over each minute of training and testing and to further evidence for within-session extinction of rats exposed to the conditioned context for 10 min. **a)** Schematic overview of experimental behavioural protocols used to generate the CONSOL, REC, and EXTNOR microarray datasets. **b)** Conditioned freezing behaviour in animals used to generate the EXTNOR Affymetrix dataset. Freezing behaviour was assessed once every 10 s. The extent of freezing is shown as number of 10 s interval units converted to percentage of max number interval units (max 6 units for 1 min, max = 12 units for 2 min). The line plots show the mean freezing values during CFC (left panel, PreUS SD (*standard deviation*) = 3.7%, PostUS SD = 26.9%) and extinction training (right panel, SD = 23.3%). There is no difference in the behaviours of the CS+ and CS- group during CFC (FREEZING X GROUP F _(2.333, 22.326) ε = 0.558_ = 1.533, P = 0.211, repeated measures ANOVA after Mauchly’s Test of Sphericity with a Greenhouse-Geisser correction). A priori repeated measures ANOVA of the separate groups during extinction training showed there was a significant effect on freezing behaviour in the CS+ but not the CS- group (CS+, F _(2.342, 11.713)_ ε _= 0.586_ = 11.034, P = 0.000 and CS-, F _(1.567, 7.824)_ ε _= 0.392_ = 1.129, P = 0.371, repeated measures ANOVA after Mauchly’s Test of Sphericity with a Greenhouse-Geisser correction). Thus, only the EXT group showed within-session extinction. **c)** Overview of experimental design for follow-up studies with qPCR (R), immunoblotting (P) and ChIP-qPCR (C). **d) and b)** Behavioural characterization of the rats for follow-up validation assays. The three rows of results represent the data from the No Recall, 2 min and 10 min recall groups, respectively. Statistical significance was tested for within-group differences (2 min vs. 10 min, paired t-test) and between-group differences between the 2min groups and 10min groups (2 min vs. 2 min and 2 min vs. 10 min, Welch’s t-test). For the 10 min Recall groups (third row) there were no FREEZING X GROUP differences (left panel CFC F _(2.009, 16.070)_ ε _= 0.502_ = 0.540, P = 0.707, right panel extinction training F _(5.474, 43.796) ε = 0.684_ = 0.621, P = 0.757, repeated measures ANOVA after Mauchly’s Test of Sphericity with a Greenhouse-Geisser correction). However, there was an effect of FREEZING during extinction training (F _(2.737, 43.796)_ ε _= 0.684_ = 10.105, P = 0.000, repeated measures ANOVA after Mauchly’s Test of Sphericity with a Greenhouse-Geisser correction), thus all groups showed a decrease in conditioned freezing behaviour across the 10 min recall session and extinction. The line plots show the mean freezing values during CFC (left panels), and recall (right panels. SD top panel, PreUS SD = 3.6%, PostUS SD = 25.9%, middle panel PreUS SD = 5.7%, PostUS SD = 32.5%, Recall SD = 23.6%, bottom panel PreUS SD = 4.6%, PostUS SD = 22.8%, Recall SD = 27.5%).
(TIF)

S1 Table Consolidation candidate gene list C1150. Genes regulated in CA1 during the consolidation of contextual fear conditioning (CONSOL-BDNFASO versus CONSOL-BDNFMSO, n = 1150 probes). Differentially expressed gene candidates are found in the 'GeneCandidates (C1150)' column marked as 'Gene candidate'. Log2 fold change ('*log2fc'), moderated t-test p-values ('*limmap'), OPLS-DA VIP ('*vip') and OPLS-DA VIPlow ('*viplow') are shown for the two normalizations (MAS and RMA). To obtain a simple traditional p-value based ranking, choose one of the two normalization types and filter/rank based on either p-value and/or log2fc sets.
(XLSX)

S2 Table Recall candidate gene list R964. Genes regulated in CA1 after the recall of contextual fear memory (REC-ZIFASO versus REC-ZIFMSO, n = 964 probes). Differentially expressed gene candidates are found in the 'GeneCandidates (R964)' column marked as 'Gene candidate'. Log2 fold change ('*log2fc'), moderated t-test p-values ('*limmap'), OPLS-DA VIP ('*vip') and OPLS-DA VIPlow ('*viplow') are shown for the two normalizations (MAS and RMA).
(XLSX)

S3 Table Extinction candidate gene list E969. Genes regulated in CA1 during the extinction of contextual fear memory (EXT versus the no recall NOR group, n = 969 probes). Differentially expressed gene candidates are found in the 'GeneCandidates (E969)' column marked as 'Gene candidate'. Log2 fold change ('*log2fc'), moderated t-test p-values ('*limmap'), OPLS-DA VIP ('*vip') and OPLS-DA VIPlow ('*viplow') are shown for the two normalizations (MAS and RMA).
(XLSX)

S4 Table Top 200 ranked genes most highly (E200ext) and lowest (E200nor) expressed in CA1 during extinction. (XLSX)

S5 Table The 120 extinction-associated genes that are clustered in enriched ontology terms. (XLSX)

S6 Table Top 200 ranked genes most highly (C200mso) and lowest (C200aso) expressed in CA1 during consolidation. (XLSX)

S7 Table Top 200 ranked genes most highly (R200mso) and lowest (R200aso) expressed in CA1 after recall. (XLSX)

S8 Table The 64 consolidation-associated genes that are clustered in enriched ontology terms. (XLSX)

S9 Table The 93 reconsolidation-associated genes that are clustered in enriched ontology terms. (XLSX)

S10 Table The candidate probes in the C1150 consolidation, R964 recall and E969 extinction datasets that were regulated after both MAS5 and RMA normalisation of the raw intensity data after hybridization (CRE1016, total of n = 1016 probes). (XLSX)

S11 Table 120 genes were up-regulated in consolidation and down-regulated in recall (profile KmeansA), and 66 genes were down-regulated in consolidation and conversely up-regulated in recall and also weakly in extinction (profile KmeansCD) after Kmeans clustering of the CRE1016 candidates. (XLSX)

## References

[1] Nader K. Memory traces unbound. Trends in neurosciences. 2003;26(2):65–72. 10.1016/S0166-2236(02)00042-5 . 12536129

[2] Pavlov IP, editor. Conditioned reflexes: An investigation of the physiological activity of the cerebral cortex London, UK: Oxford University Press; 1927. 10.5214/ans.0972-7531.1017309 PMC4116985 25205891

[3] Rescorla RA. Retraining of extinguished Pavlovian stimuli. Journal of experimental psychology Animal behavior processes. 2001;27(2):115–24. . 11296487

[4] Myers KM, Carlezon WA Jr, Davis M. Glutamate receptors in extinction and extinction-based therapies for psychiatric illness. Neuropsychopharmacology: official publication of the American College of Neuropsychopharmacology. 2011;36(1):274–93. 10.1038/npp.2010.88 20631689 PMC2994960

[5] Milad MR, Quirk GJ. Fear extinction as a model for translational neuroscience: ten years of progress. Annual review of psychology. 2012;63:129–51. 10.1146/annurev.psych.121208.131631 . 22129456 PMC4942586

[6] Johansen JP, Wolff SB, Luthi A, LeDoux JE. Controlling the elements: an optogenetic approach to understanding the neural circuits of fear. Biological psychiatry. 2012;71(12):1053–60. 10.1016/j.biopsych.2011.10.023 22169096 PMC3319499

[7] Lee JL, Everitt BJ, Thomas KL. Independent cellular processes for hippocampal memory consolidation and reconsolidation. Science. 2004;304(5672):839–43. 10.1126/science.1095760 . 15073322

[8] Tronson NC, Taylor JR. Molecular mechanisms of memory reconsolidation. Nature reviews Neuroscience. 2007;8(4):262–75. 10.1038/nrn2090 . 17342174

[9] Barnes P, Thomas KL. Proteolysis of proBDNF is a key regulator in the formation of memory. PloS one. 2008;3(9):e3248 10.1371/journal.pone.0003248 18813339 PMC2532744

[10] Pedreira ME, Maldonado H. Protein synthesis subserves reconsolidation or extinction depending on reminder duration. Neuron. 2003;38(6):863–9. . 12818173 10.1016/s0896-6273(03)00352-0

[11] Eisenberg M, Kobilo T, Berman DE, Dudai Y. Stability of retrieved memory: inverse correlation with trace dominance. Science. 2003;301(5636):1102–4. 10.1126/science.1086881 . 12934010

[12] Suzuki A, Josselyn SA, Frankland PW, Masushige S, Silva AJ, Kida S. Memory reconsolidation and extinction have distinct temporal and biochemical signatures. The Journal of neuroscience: the official journal of the Society for Neuroscience. 2004;24(20):4787–95. 10.1523/JNEUROSCI.5491-03.2004 . 15152039 PMC6729467

[13] Power AE, Berlau DJ, McGaugh JL, Steward O. Anisomycin infused into the hippocampus fails to block "reconsolidation" but impairs extinction: the role of re-exposure duration. Learning & memory. 2006;13(1):27–34. 10.1101/lm.91206 16452651 PMC1360130

[14] Inda MC, Muravieva EV, Alberini CM. Memory retrieval and the passage of time: from reconsolidation and strengthening to extinction. The Journal of neuroscience: the official journal of the Society for Neuroscience. 2011;31(5):1635–43. 10.1523/JNEUROSCI.4736-10.2011 21289172 PMC3069643

[15] Perez-Cuesta LM, Maldonado H. Memory reconsolidation and extinction in the crab: mutual exclusion or coexistence? Learning & memory. 2009;16(11):714–21. 10.1101/lm.1544609 . 19875505

[16] Bouton ME, Westbrook RF, Corcoran KA, Maren S. Contextual and temporal modulation of extinction: behavioral and biological mechanisms. Biological psychiatry. 2006;60(4):352–60. 10.1016/j.biopsych.2005.12.015 . 16616731

[17] Ben Mamou C, Gamache K, Nader K. NMDA receptors are critical for unleashing consolidated auditory fear memories. Nature neuroscience. 2006;9(10):1237–9. 10.1038/nn1778 . 16998481

[18] Lee JL, Everitt BJ. Reactivation-dependent amnesia in Pavlovian approach and instrumental transfer. Learning & memory. 2008;15(8):597–602. 10.1101/lm.1029808 18685151 PMC2583130

[19] Suzuki A, Mukawa T, Tsukagoshi A, Frankland PW, Kida S. Activation of LVGCCs and CB1 receptors required for destabilization of reactivated contextual fear memories. Learning & memory. 2008;15(6):426–33. 10.1101/lm.888808 18511694 PMC2414253

[20] Lee SH, Choi JH, Lee N, Lee HR, Kim JI, Yu NK, et al Synaptic protein degradation underlies destabilization of retrieved fear memory. Science. 2008;319(5867):1253–6. 10.1126/science.1150541 . 18258863

[21] Boccia M, Freudenthal R, Blake M, de la Fuente V, Acosta G, Baratti C, et al Activation of hippocampal nuclear factor-kappa B by retrieval is required for memory reconsolidation. The Journal of neuroscience: the official journal of the Society for Neuroscience. 2007;27(49):13436–45. 10.1523/JNEUROSCI.4430-07.2007 . 18057202 PMC6673108

[22] Hall J, Thomas KL, Everitt BJ. Cellular imaging of zif268 expression in the hippocampus and amygdala during contextual and cued fear memory retrieval: selective activation of hippocampal CA1 neurons during the recall of contextual memories. The Journal of neuroscience: the official journal of the Society for Neuroscience. 2001;21(6):2186–93. . 11245703 10.1523/JNEUROSCI.21-06-02186.2001 PMC6762622

[23] Lin CH, Yeh SH, Lu HY, Gean PW. The similarities and diversities of signal pathways leading to consolidation of conditioning and consolidation of extinction of fear memory. The Journal of neuroscience: the official journal of the Society for Neuroscience. 2003;23(23):8310–7. . 12967993 10.1523/JNEUROSCI.23-23-08310.2003 PMC6740702

[24] Strekalova T, Zorner B, Zacher C, Sadovska G, Herdegen T, Gass P. Memory retrieval after contextual fear conditioning induces c-Fos and JunB expression in CA1 hippocampus. Genes, brain, and behavior. 2003;2(1):3–10. . 12882314 10.1034/j.1601-183x.2003.00001.x

[25] Romano A, Locatelli F, Freudenthal R, Merlo E, Feld M, Ariel P, et al Lessons from a crab: molecular mechanisms in different memory phases of Chasmagnathus. The Biological bulletin. 2006;210(3):280–8. . 16801501 10.2307/4134564

[26] Fischer A, Radulovic M, Schrick C, Sananbenesi F, Godovac-Zimmermann J, Radulovic J. Hippocampal Mek/Erk signaling mediates extinction of contextual freezing behavior. Neurobiology of learning and memory. 2007;87(1):149–58. 10.1016/j.nlm.2006.08.003 16979915 PMC1839930

[27] Sananbenesi F, Fischer A, Wang X, Schrick C, Neve R, Radulovic J, et al A hippocampal Cdk5 pathway regulates extinction of contextual fear. Nature neuroscience. 2007;10(8):1012–9. 10.1038/nn1943 17632506 PMC2441763

[28] Malvaez M, McQuown SC, Rogge GA, Astarabadi M, Jacques V, Carreiro S, et al HDAC3-selective inhibitor enhances extinction of cocaine-seeking behavior in a persistent manner. Proceedings of the National Academy of Sciences of the United States of America. 2013;110(7):2647–52. 10.1073/pnas.1213364110 23297220 PMC3574934

[29] Corlett PR, Krystal JH, Taylor JR, Fletcher PC. Why do delusions persist? Frontiers in human neuroscience. 2009;3:12 10.3389/neuro.09.012.2009 19636384 PMC2713737

[30] Holt DJ, Lebron-Milad K, Milad MR, Rauch SL, Pitman RK, Orr SP, et al Extinction memory is impaired in schizophrenia. Biological psychiatry. 2009;65(6):455–63. 10.1016/j.biopsych.2008.09.017 . 18986648 PMC3740529

[31] Tronson NC, Taylor JR. Addiction: a drug-induced disorder of memory reconsolidation. Current opinion in neurobiology. 2013 10.1016/j.conb.2013.01.022 . 23415831 PMC3677957

[32] Parsons RG, Ressler KJ. Implications of memory modulation for post-traumatic stress and fear disorders. Nature neuroscience. 2013;16(2):146–53. 10.1038/nn.3296 . 23354388 PMC3752300

[33] Schiller D, Monfils MH, Raio CM, Johnson DC, Ledoux JE, Phelps EA. Preventing the return of fear in humans using reconsolidation update mechanisms. Nature. 2010;463(7277):49–53. 10.1038/nature08637 20010606 PMC3640262

[34] Kindt M, Soeter M. Reconsolidation in a human fear conditioning study: a test of extinction as updating mechanism. Biological psychology. 2013;92(1):43–50. 10.1016/j.biopsycho.2011.09.016 . 21986472

[35] Soeter M, Kindt M. Dissociating response systems: erasing fear from memory. Neurobiology of learning and memory. 2010;94(1):30–41. 10.1016/j.nlm.2010.03.004 . 20381628

[36] Barnes P, Kirtley A, Thomas KL. Quantitatively and qualitatively different cellular processes are engaged in CA1 during the consolidation and reconsolidation of contextual fear memory. Hippocampus. 2012;22(2):149–71. 10.1002/hipo.20879 . 21080409

[37] Merlo E, Romano A. Memory extinction entails the inhibition of the transcription factor NF-kappaB. PloS one. 2008;3(11):e3687 10.1371/journal.pone.0003687 18997870 PMC2577885

[38] de la Fuente V, Freudenthal R, Romano A. Reconsolidation or extinction: transcription factor switch in the determination of memory course after retrieval. The Journal of neuroscience: the official journal of the Society for Neuroscience. 2011;31(15):5562–73. 10.1523/JNEUROSCI.6066-10.2011 . 21490196 PMC6622842

[39] Chang YJ, Yang CH, Liang YC, Yeh CM, Huang CC, Hsu KS. Estrogen modulates sexually dimorphic contextual fear extinction in rats through estrogen receptor beta. Hippocampus. 2009;19(11):1142–50. 10.1002/hipo.20581 . 19338017

[40] Gerstner JR, Yin JC. Circadian rhythms and memory formation. Nature reviews Neuroscience. 2010;11(8):577–88. 10.1038/nrn2881 . 20648063 PMC6544049

[41] Panja D, Bramham CR. BDNF mechanisms in late LTP formation: A synthesis and breakdown. Neuropharmacology. 2014;76 Pt C:664–76. 10.1016/j.neuropharm.2013.06.024 . 23831365

[42] Smyth GK. Linear models and empirical bayes methods for assessing differential expression in microarray experiments. Statistical applications in genetics and molecular biology. 2004;3:Article3. 10.2202/1544-6115.1027 . 16646809

[43] Bylesjö M RM, Cloarec O, Nicholson JK, Holmes E, Trygg J. OPLS discriminant analysis: combining the strengths of PLS-DA and SIMCA classification. Journal of Chemometrics. 2006;20:341–51.

[44] Huang DW, Sherman BT, Lempicki RA. Systematic and integrative analysis of large gene lists using DAVID bioinformatics resources. Nature Protocols. 2009;4:44–57. 10.1038/nprot.2008.211 19131956

[45] Loots GG, Ovcharenko I. rVISTA 2.0: evolutionary analysis of transcription factor binding sites. Nucleic Acids Research. 2004;32:W217–21. 15215384 10.1093/nar/gkh383 PMC441521

[46] Dubchak I, Poliakov A, Kislyuk A, Brudno M. Multiple whole-genome alignments without a reference organism. Genome research. 2009;19(4):682–9. 10.1101/gr.081778.108 19176791 PMC2665786

[47] Plendl W, Wotjak CT. Dissociation of within- and between-session extinction of conditioned fear. The Journal of neuroscience: the official journal of the Society for Neuroscience. 2010;30(14):4990–8. 10.1523/JNEUROSCI.6038-09.2010 . 20371819 PMC6632794

[48] Edgar R, Domrachev M, Lash AE. Gene Expression Omnibus: NCBI gene expression and hybridization array data repository. Nucleic Acids Res. 2002;30(1):207–10. 11752295 10.1093/nar/30.1.207 PMC99122

[49] Merlo E, Freudenthal R, Maldonado H, Romano A. Activation of the transcription factor NF-kappaB by retrieval is required for long-term memory reconsolidation. Learning & memory. 2005;12(1):23–9. 10.1101/lm.82705 15687229 PMC548492

[50] Hall J, Thomas KL, Everitt BJ. Rapid and selective induction of BDNF expression in the hippocampus during contextual learning. Nature neuroscience. 2000;3(6):533–5. 10.1038/75698 . 10816306

[51] Pickering M, Cumiskey D, O'Connor JJ. Actions of TNF-alpha on glutamatergic synaptic transmission in the central nervous system. Experimental physiology. 2005;90(5):663–70. 10.1113/expphysiol.2005.030734 . 15944202

[52] Tonelli LH, Postolache TT, Sternberg EM. Inflammatory genes and neural activity: involvement of immune genes in synaptic function and behavior. Frontiers in bioscience: a journal and virtual library. 2005;10:675–80. . 15569608 10.2741/1562

[53] Stellwagen D, Malenka RC. Synaptic scaling mediated by glial TNF-alpha. Nature. 2006;440(7087):1054–9. 10.1038/nature04671 . 16547515

[54] Dalton GL, Wang YT, Floresco SB, Phillips AG. Disruption of AMPA receptor endocytosis impairs the extinction, but not acquisition of learned fear. Neuropsychopharmacology: official publication of the American College of Neuropsychopharmacology. 2008;33(10):2416–26. 10.1038/sj.npp.1301642 . 18046303

[55] Migues PV, Hardt O, Wu DC, Gamache K, Sacktor TC, Wang YT, et al PKMzeta maintains memories by regulating GluR2-dependent AMPA receptor trafficking. Nature neuroscience. 2010;13(5):630–4. 10.1038/nn.2531 . 20383136

[56] Rao-Ruiz P, Rotaru DC, van der Loo RJ, Mansvelder HD, Stiedl O, Smit AB, et al Retrieval-specific endocytosis of GluA2-AMPARs underlies adaptive reconsolidation of contextual fear. Nature neuroscience. 2011;14(10):1302–8. 10.1038/nn.2907 . 21909089

[57] Monfils MH, Cowansage KK, Klann E, LeDoux JE. Extinction-reconsolidation boundaries: key to persistent attenuation of fear memories. Science. 2009;324(5929):951–5. 10.1126/science.1167975 19342552 PMC3625935

[58] Lee JL. Memory reconsolidation mediates the updating of hippocampal memory content. Frontiers in behavioral neuroscience. 2010;4:168 10.3389/fnbeh.2010.00168 21120142 PMC2991235

[59] Morris RG, Inglis J, Ainge JA, Olverman HJ, Tulloch J, Dudai Y, et al Memory reconsolidation: sensitivity of spatial memory to inhibition of protein synthesis in dorsal hippocampus during encoding and retrieval. Neuron. 2006;50(3):479–89. 10.1016/j.neuron.2006.04.012 . 16675401

[60] Krieglstein K, Zheng F, Unsicker K, Alzheimer C. More than being protective: functional roles for TGF-beta/activin signaling pathways at central synapses. Trends in neurosciences. 2011;34(8):421–9. 10.1016/j.tins.2011.06.002 . 21742388

[61] Ageta H, Ikegami S, Miura M, Masuda M, Migishima R, Hino T, et al Activin plays a key role in the maintenance of long-term memory and late-LTP. Learning & memory. 2010;17(4):176–85. 10.1101/lm.16659010 . 20332189

[62] Zhang F, Endo S, Cleary LJ, Eskin A, Byrne JH. Role of transforming growth factor-beta in long-term synaptic facilitation in Aplysia. Science. 1997;275(5304):1318–20. . 9036859 10.1126/science.275.5304.1318

[63] Fukushima T, Liu RY, Byrne JH. Transforming growth factor-beta2 modulates synaptic efficacy and plasticity and induces phosphorylation of CREB in hippocampal neurons. Hippocampus. 2007;17(1):5–9. 10.1002/hipo.20243 . 17094084

[64] Nguyen PT, Nakamura T, Hori E, Urakawa S, Uwano T, Zhao J, et al Cognitive and socio-emotional deficits in platelet-derived growth factor receptor-beta gene knockout mice. PloS one. 2011;6(3):e18004 10.1371/journal.pone.0018004 21437241 PMC3060876

[65] Shioda N, Moriguchi S, Oya T, Ishii Y, Shen J, Matsushima T, et al Aberrant hippocampal spine morphology and impaired memory formation in neuronal platelet-derived growth factor beta-receptor lacking mice. Hippocampus. 2012;22(6):1371–8. 10.1002/hipo.20973 . 21997856

[66] Lubin FD, Sweatt JD. The IkappaB kinase regulates chromatin structure during reconsolidation of conditioned fear memories. Neuron. 2007;55(6):942–57. 10.1016/j.neuron.2007.07.039 17880897 PMC2587178

[67] Li Q, Verma IM. NF-kappaB regulation in the immune system. Nature reviews Immunology. 2002;2(10):725–34. 10.1038/nri910 . 12360211

[68] McAfoose J, Baune BT. Evidence for a cytokine model of cognitive function. Neuroscience and biobehavioral reviews. 2009;33(3):355–66. 10.1016/j.neubiorev.2008.10.005 . 18996146

[69] Trent S, Branes P, Hall J, Thomas KL. Rescue of long-term memory after reconsolidation blockade. Nature communications. 2015;6:7897 Epub 4 August 2015. 10.1038/ncomms8897 26238574 PMC4532853

[70] Kim MY, Jeong BC, Lee JH, Kee HJ, Kook H, Kim NS, et al A repressor complex, AP4 transcription factor and geminin, negatively regulates expression of target genes in nonneuronal cells. Proceedings of the National Academy of Sciences of the United States of America. 2006;103(35):13074–9. 10.1073/pnas.0601915103 16924111 PMC1551900

[71] Hong SJ, Choi HJ, Hong S, Huh Y, Chae H, Kim KS. Transcription factor GATA-3 regulates the transcriptional activity of dopamine beta-hydroxylase by interacting with Sp1 and AP4. Neurochemical research. 2008;33(9):1821–31. 10.1007/s11064-008-9639-3 18338249 PMC2712938

[72] Kirtley A, Thomas KL. The exclusive induction of extinction is gated by BDNF. Learning & memory. 2010;17(12):612–9. 10.1101/lm.1877010 . 21127000

[73] Baumgartel K, Genoux D, Welzl H, Tweedie-Cullen RY, Koshibu K, Livingstone-Zatchej M, et al Control of the establishment of aversive memory by calcineurin and Zif268. Nature neuroscience. 2008;11(5):572–8. 10.1038/nn.2113 . 18425121

[74] Mitchell N, Petralia RS, Currier DG, Wang YX, Kim A, Mattson MP, et al Sonic hedgehog regulates presynaptic terminal size, ultrastructure and function in hippocampal neurons. Journal of cell science. 2012;125(Pt 18):4207–13. 10.1242/jcs.105080 22641692 PMC3516435

[75] Harwell CC, Parker PR, Gee SM, Okada A, McConnell SK, Kreitzer AC, et al Sonic hedgehog expression in corticofugal projection neurons directs cortical microcircuit formation. Neuron. 2012;73(6):1116–26. 10.1016/j.neuron.2012.02.009 22445340 PMC3551478

[76] Millan MJ. An epigenetic framework for neurodevelopmental disorders: from pathogenesis to potential therapy. Neuropharmacology. 2013;68:2–82. 10.1016/j.neuropharm.2012.11.015 . 23246909

[77] Quirk GJ, Mueller D. Neural mechanisms of extinction learning and retrieval. Neuropsychopharmacology: official publication of the American College of Neuropsychopharmacology. 2008;33(1):56–72. 10.1038/sj.npp.1301555 17882236 PMC2668714

