# Supplementary figures and images for: The Regulation of Cytokine Networks in Hippocampal CA1 Differentiates Extinction from Those Required for the Maintenance of Contextual Fear Memory after Recall
Source: PLoS One. 2016 May 25;11(5):e0153102. doi: 10.1371/journal.pone.0153102 (PMC4880201; doi:10.1371/journal.pone.0153102)

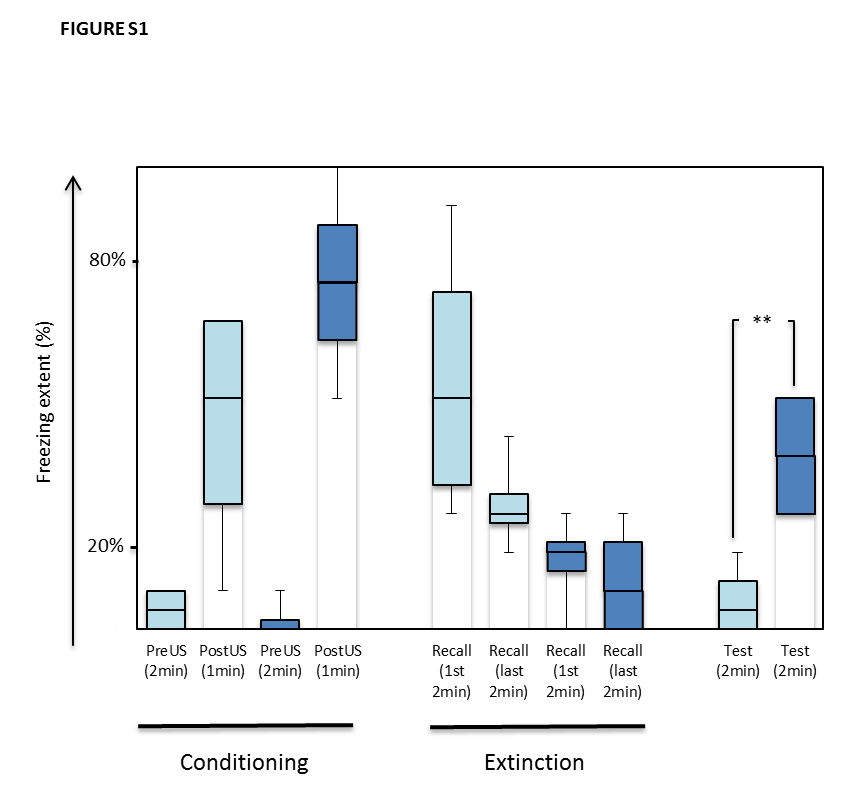

Supplement: S1 Fig — Rats were first pre-exposed for 3 days to two experimental chambers for 10 min/d. These contexts differed in a number of characteristics including size, spatial location, odor and lighting, and exposure to each chamber was separated by a minimum of 4 hours. The conditioning trial was given 24 hours later. Conditioning consisted of the rats being placed individually in one of the chambers (at the same time of day as during the pre-exposure sessions) for 3 min in a counterbalanced manner and a single scrambled foot shock (US, 0.5 mA for 2 s) was delivered at 2 min. The rats were then returned to their home cages. Forty-eight hours later, rats were either returned to the CS conditioned context (CS+, EXT group, light blue, n = 4) or to the other context (CS- no fear recall, NOR group, mid blue, n = 4) for 10 min. All rats were subsequently exposed to the CS+ for 2 min (Test) 48 hours later. Rats showed an increase in postUS freezing, however conditioned freezing behaviour was only seen in the EXT group exposed CS+ during the first two min of the 10 min non-reinforced extinction training (i.e. a difference in behaviour between the PostUS and Recall (1st 2 min) was seen for the NOR group only, P < 0.01). There was a trend towards a decrease in conditioned freezing measured in the last two min of the extinction in this group (within-session extinction which represents extinction memory acquisition[77]). The EXT group also showed less conditioned fear to the CS+ during the Test than the NOR group. Thus, exposure to the CS+ for 10 min is necessary for a persistent reduction in conditioned fear behaviour (between-session extinction). Statistical significance was tested for within-group differences (paired t-test) and between-group differences at test (Welch’s t-test). **P < 0.01. (TIF) [file pone.0153102.s001.tif]

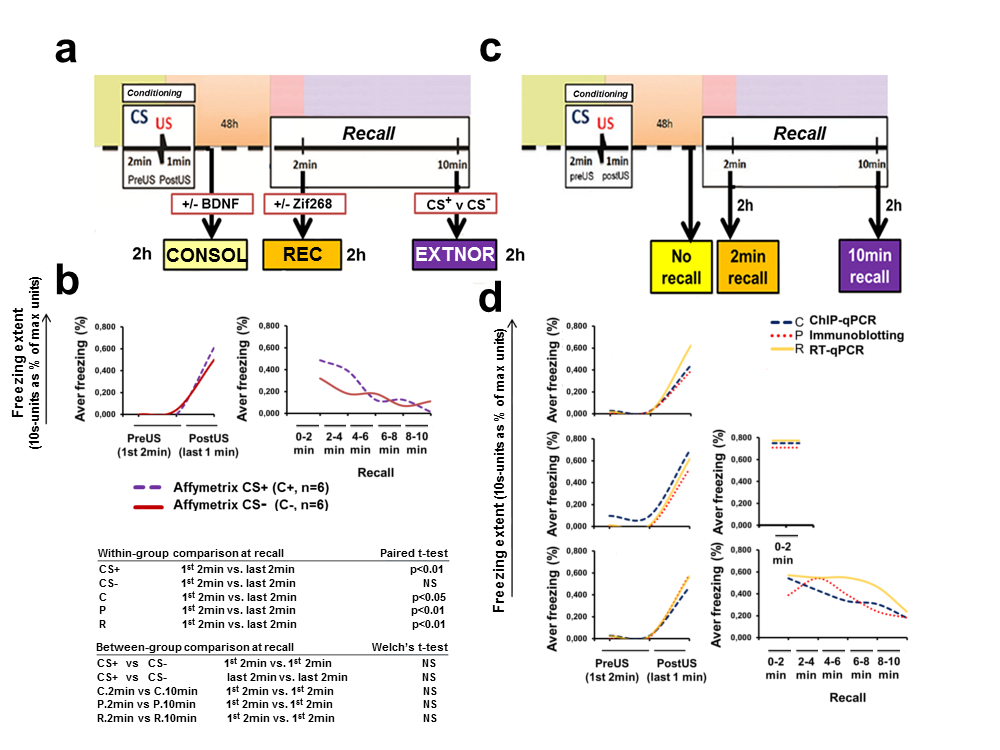

Supplement: S2 Fig — a) Schematic overview of experimental behavioural protocols used to generate the CONSOL, REC, and EXTNOR microarray datasets. b) Conditioned freezing behaviour in animals used to generate the EXTNOR Affymetrix dataset. Freezing behaviour was assessed once every 10 s. The extent of freezing is shown as number of 10 s interval units converted to percentage of max number interval units (max 6 units for 1 min, max = 12 units for 2 min). The line plots show the mean freezing values during CFC (left panel, PreUS SD (standard deviation) = 3.7%, PostUS SD = 26.9%) and extinction training (right panel, SD = 23.3%). There is no difference in the behaviours of the CS+ and CS- group during CFC (FREEZING X GROUP F (2.333, 22.326) ε = 0.558 = 1.533, P = 0.211, repeated measures ANOVA after Mauchly’s Test of Sphericity with a Greenhouse-Geisser correction). A priori repeated measures ANOVA of the separate groups during extinction training showed there was a significant effect on freezing behaviour in the CS+ but not the CS- group (CS+, F (2.342, 11.713) ε = 0.586 = 11.034, P = 0.000 and CS-, F (1.567, 7.824) ε = 0.392 = 1.129, P = 0.371, repeated measures ANOVA after Mauchly’s Test of Sphericity with a Greenhouse-Geisser correction). Thus, only the EXT group showed within-session extinction. c) Overview of experimental design for follow-up studies with qPCR (R), immunoblotting (P) and ChIP-qPCR (C). d) and b) Behavioural characterization of the rats for follow-up validation assays. The three rows of results represent the data from the No Recall, 2 min and 10 min recall groups, respectively. Statistical significance was tested for within-group differences (2 min vs. 10 min, paired t-test) and between-group differences between the 2min groups and 10min groups (2 min vs. 2 min and 2 min vs. 10 min, Welch’s t-test). For the 10 min Recall groups (third row) there were no FREEZING X GROUP differences (left panel CFC F (2.009, 16.070) ε = 0.502 = 0.540, P = 0.707, right panel extinction [file pone.0153102.s002.tif]
